# Antarctic Krill with parasites grow slower than uninfected peers

**DOI:** 10.1007/s00227-025-04673-w

**Published:** 2025-06-11

**Authors:** A. C. Cleary, S. Kawaguchi, R. King, J. E. Melvin, G. A. Tarling

**Affiliations:** 1 British Antarctic Survey, Cambridge, UK; 2 Australian Antarctic Division, Kingston, Australia; 3 Australian Antarctic Program Partnership, Hobart, Australia; 4 Institute for Marine and Antarctic Science, University of Tasmania, Hobart, Australia

**Keywords:** Parasite, *Euphausia superba*, 18S metabarcoding, Apicomplexa, Ciliate, East Antarctica

## Abstract

Antarctic krill, *Euphausia superba* (Dana i Am J Sci Arts Ser 2(9):129–133, 1850), are both central ecosystem components in the Southern Ocean, and the target of a growing commercial fishery. Understanding the trophic interactions which shape krill population dynamics is essential to sustainably managing human impacts on this key species. While the roles of krill as grazers of phytoplankton and as prey for vertebrate predators are relatively well understood, very little is known about interactions with their smallest predators– the parasites. We investigated the assemblage of parasites present in *E. superba*, and the impacts of parasites on krill somatic growth. We found 15 distinct parasite types across a sample of 100 krill, including seven types of Gregarine Apicomplexa, two types of internally infecting ciliates, three types of epibiotic ciliates, fungi, syndiniales, and a *Parorchites zederi* cestode worm. Apicomplexa and epibiotic ciliate infections were linked with lower growth rates, with these two parasites explaining 10% and 24% of the observed variation in krill growth, respectively. Although much uncertainty remains, scaling these results to the population level suggests parasites may be responsible for as much reduction in krill biomass annually as vertebrate predators, indicating the importance of considering these trophic links in food web modelling and ecosystem-based management.

**Supplementary Information:**

The online version contains supplementary material available at 10.1007/s00227-025-04673-w.

## Introduction

Trophic interactions form the scaffold of ecosystem structure, determining how energy and organic matter flow through ecosystems (Murphy et al. 2016). Compared to grazing and predation, parasitism is a class of trophic interactions which has received disproportionately little attention, particularly in natural marine ecosystems and within the lower trophic level organisms (Lafferty et al. 2008). Yet, when parasites have been carefully quantified in marine systems, their biomass has been found to exceed that of vertebrate top predators, and parasite-host interactions have been the most common type of trophic links (Lafferty et al. 2006; Kuris et al. 2008). This suggests that parasites may play significant roles in marine ecosystems, potentially influencing the flows of carbon and energy, as well as impacting populations of their hosts with potential ripple effects onto the prey, predators, and competitors of these hosts (Bennet et al. 2024).

Understanding the role of parasites is particularly important in managing the impacts of human activities under a changing environment. The environment is shifting due to anthropogenic climate change, with predicted changes in temperature, circulation, and primary production. These shifts are likely to impact the balances between parasites and hosts, and may also drive evolutionary changes within parasite species (Cable et al. 2017; Poulin et al. 2024). Ignoring these trophic interactions may limit the effectiveness of sustainability measures, such as fisheries policies (Dobson and May 1987; Lafferty and Kuris 1999; Marcogliese 2004).

The Southern Ocean is both experiencing rapid environmental changes due to climate and an increase in fisheries pressure. Antarctic krill, *Euphausia superba*, are the main target of the growing international fishery (Kawaguchi and Nicol 2020; CCAMLR Secretariat 2022). These small crustaceans play key ecosystem roles, serving as the main prey item for most of the vertebrate predators of the region, including seals, whales, penguins, and flying seabirds (Quetin and Ross 1991; Trathan and Hill 2016). *Euphausia superba* are also active in carbon cycling and the biological carbon pump, helping to export carbon to the deep sea through both vertical migration and the production of rapidly sinking faecal pellets (Le Fèvre et al. 1998).

*Euphausia superba* are known to host a diverse range of pathogenic, parasitic, and parasitoid organisms, although the frequency of infection and geographic ranges of these organisms remain largely unknown. Eukaryotic symbionts of *E. superba* include four broad groups of single celled protists - apicomplexa gut parasites, ciliates attached to krill’s exoskeleton, ciliates within the krill body cavity, and fungi, as well as two groups of metazoans– isopods and nematodes (Gómez-Gutiérrez and Morales-Ávila 2016). *Euphausia superba* spend most of their lives in dense schools or swarms, a behaviour which may increase their susceptibility to parasites and pathogens (Burns et al. 2022).

The impact of parasite infection on krill individuals and populations also remains uncertain. Parasite impacts on pelagic crustaceans range from lethal (parasitoids), to no discernible effect, with a wide range intensities and mechanisms in between (Skovgaard 2014). Parasites may take energy directly from host tissues, take energy from partially digested prey within the gut, or use the host simply as a mobile platform, with indirect effects such as increased drag or increased visibility to predators (Skovgaard 2014). Of the described parasites of Antarctic krill, the *Pseudocollinia* spp. ciliates are considered to have the largest impact at the level of the individual host– multiplying within the krill’s body and consuming its resources to the point of mortality (Gómez-Gutiérrez et al. 2003). It has been suggested these ciliates may be responsible for mass mortality events which have been observed in *E. superba*, but most of what is known about these *Pseudocollinia* spp. infections comes from other euphausiid species (Lynn et al. 2014; Gómez-Gutiérrez and Kawaguchi 2017). Apicomplexa parasites are thought to reduce the efficiency of digestion and decrease the amount of nutrients krill can obtain from ingested prey, as well as reducing the function of the liver (Avdeev and Vagin 1987; Takahashi et al. 2008). Epibiotic ciliates have been suggested to exert a cost on krill by increasing hydrodynamic drag (Buchholz et al. 2006). Both ciliate and Apicomplexa infections have been associated with krill producing poorly resourced eggs, potentially impacting larval survival and eventual recruitment success (Cleary et al. 2024). Culturing poorly known parasitic organisms is challenging and there are currently no publications reporting experimentally infected euphausiids; making assessing the impacts of parasites on *E. superba* particularly difficult.

In this study we investigated the impact of natural parasite infections on somatic growth in *E. superba*. Growth is a key component of secondary production– generating the biomass available to higher trophic levels and commercial fisheries. *Euphausia superba* growth is influenced by environmental factors including temperature and the availability of phytoplankton prey, but these factors alone typically explain less than half of the observed variation in growth rates (Atkinson et al. 2006). We here combined incubation experiments measuring near in-situ growth rates with high sensitivity genetic detection of all eukaryotic parasites within and on *E. superba* to determine (A) the assemblage of parasites infecting *E. superba* in East Antarctica and (B) the impact of parasite infection on *E. superba* somatic growth. Combining parasite infection prevalences and individual level impacts allows for first insights into the potential population and ecosystem effects of these understudied trophic links in the Southern Ocean.

## Materials and methods

*Euphausia superba* were collected and incubated on the TEMPO (Trends in Euphausiids off Mawson, Predators, and Oceanography) voyage of RV Investigator between February 20 th and March 9 th, 2021. Krill were collected by targeting krill-like echoes on vessel’s echo sounder with a rectangular mid-water trawl (RMT8 + 1) with an 8 m^2^ mouth opening and mesh size of 4.5 mm. All tows were conducted in the upper 70 m of the water column. Environmental data (depth, temperature, salinity, chlorophyll fluorescence) were collected during these tows with an RBRconcerto conductivity, temperature, and depth instrument package (CTD) attached to the net frame, with values averaged over the net open interval for analyses (Table 1).

**Table 1:** Environmental parameters during sampling for each of the analysed IGR incubations. time is the Tow midpoint in universal time coordinated (UTC)

Experiment	Date (2021)	Time UTC (mean)	Latitude (mean)	Longitude (mean)	Bottom depth	Temp (mean)	Chla (mean)
1	Feb-20	06:05	−66.03	60.03	ND	−1.32	0.60
2	Feb-21	06:58	−66.58	62.08	773	−1.66	0.27
3	Feb-24	04:28	−66.01	64.99	2456	−1.29	0.67
9	Mar-06	19:32	−66.47	75.25	2429	−0.45	6.32
11	Mar-09	23:47	−63.00	79.78	3566	0.88	0.22

Instantaneous growth rate (IGR) experiments were performed as described in Kawaguchi et al. (2006). In brief, individual krill were gently transferred into 500 ml perforated jars within a 1000 l tank of continuous flowing surface seawater. Krill jars were checked every 12 h to determine if each krill had moulted, for a maximum of four days. The maximum length of incubation is consistent with other IGR studies, and represents a compromise between capturing sufficient moulting events, while minimizing the time krill are held under aquarium conditions which never perfectly replicate those in-situ (Tarling et al. 2006). When a krill moulted, both moult and krill were frozen for return to shore. After returning to land, total length and uropod length was measured for both krill and moults as per Atkinson et al. (2006), and samples were re-frozen until genetic analysis. Growth rate was calculated using the average of both left and right uropods, as per Atkinson et al. (2006).

Genetic analysis was conducted on a subset of the incubated krill– including the ten highest and lowest growth rate individuals from each of five stations in order to capture both a range of growth rates and a range of environmental conditions. The total number of krill which moulted in each IGR experiment ranged from 56 to 94; a subsample for genetics of 20 of these thus represents 21–35% of the total moulted individuals from each experiment. Samples were chosen without regard to days-before-moulting or individual length, to select as representative a subset as possible given that initial analyses did not show strong correlations between these two factors and growth rate. Genomic DNA was extracted from each krill and corresponding moult using the DNeasy blood and tissue kit (Qiagen). Both bodies and moults were analyzed as moulting is thought to remove many parasites colonizing the exterior of the krill and the gut lining, and thus shed moults may give a more representative picture of the parasite load experienced by a krill. In order to capture a representative sample of DNA, krill were first individually homogenized in 2 ml of TNES buffer (Tris, Sodium Chloride, EDTA, and SDS) with a tissue blender, and a sub-sample of 200 µl carried forward, while moults were manually broken up with a pipette tip in 200 µl of TNES buffer. For both krill and moult samples, these initial 200 µl TNES slurries were then combined with 200 µl of buffer ATL, and digested with 40 µl of Proteinase K at 56^o^, then centrifuged to remove any residual undigested chitin, and 220 µl of the digest carried forward in the extraction kit, with all remaining steps as per manufacturer’s directions.

18 S ribosomal DNA variable region 7 was amplified from all non-krill DNA using a nested Peptide Nucleic Acid Polymerase Chain Reaction approach with universal eukaryote primers as per Cleary et al. (2024) (forward - CGGCTYAATTYGAYTCAACRC, reverse GGGCATCACRGACCTG) with second round primers modified to include sequencing-centre-specifc adaptors.

The choice of genetic marker is always a trade-off between coverage and resolution; we prioritized capturing the full range of eukaryotic parasites potentially present in krill, and note that the commonly used barcoding gene CoI is absent from the key parasite group of marine apicomplexa (Salomaki et al. 2021). Each PCR set included an amplification no template control, all of which were confirmed to contain no detectible DNA on a high sensitivity bioanalyzer chip. Two extraction-PCR-sequencing no template controls were carried through the complete laboratory processing and included in the final sequencing. Amplicons were barcoded and sequenced on a 2 × 300 run of an Illumina MiSeq by the Ramaciotti Centre for Genomics (Sydney, Australia) following their standard protocols.

Bioinformatics were conducted in Qiime2 on the British Antarctic Survey linux high performance computing cluster (Bolyen et al. 2019). Forward and reverse primers were trimmed from each read using cut-adapt, and any reads lacking the correct forward primer sequence were discarded (Martin 2011). Filtering was more lenient for reverse reads, as strict trimming could bias against parasitic organisms which are more likely to have DNA inserts, affecting the total amplicon length (Bromham et al. 2013). Reads were further quality controlled, paired, and grouped into exact amplicon sequence variants (ASVs) in dada2 (Callahan et al. 2016). The taxonomic affiliation of each sequence was categorized using a SciKit-learn machine learning algorithm trained on the Silva v. 138 database trimmed to contain only this V7 amplicon, and refined using BLAST searches against the complete GenBank database in cases where automated taxonomy results were ambiguous (Altschul et al. 1990; Pedregosa et al. 2011; Boratyn et al. 2013). ASVs which could not be classified more finely than eukaryote were excluded from analysis as they are not interpretable (158,400 reads, 0.46% of total), as were a handful of ASVs associated with organisms not expected to be present in the marine environment, as these likely represent errors or contaminants (20,393 reads, 0.06% of total). A final quality control filtering step removed ASVs with a total sequence read abundance of less than 0.01% of the overall total, as these rarest sequences often contain errors and increase processing time significantly without adding to the overall interpretation (combined 3,765,577 reads, 10.88% or total).

ASVs were categorized as krill, parasite, or prey. ASVs taxonomically identified as *E. superba*, or which could only be confidently classified at a broader taxonomic level and fell in any category which includes *E. superba* such as “crustacea”, were considered as krill. ASVs which were confidently identified as belonging to a group which had previously been reported as parasites were considered as parasites. For some taxonomic groups, this distinction is ambiguous, as parasitic lifestyles are found in close relatives to free living organisms, in which case we aimed for a conservative approach. For fungi, the parasitic groups included the orders Hypocreales (Leaño 2001; Pang et al. 2021) and Chaetothyriales, the family Metschnikoviacea, and the genus *Aspergillus*. For ciliates, the families Oligohymenophorea, Phyllopharynea, and Haptoria and the genus *Ephelota* were considered as parasitic (Morado and Small 1995). The remaining ASVs consisted of various planktonic organisms, and were considered as prey. Additional quality control of parasite ASVs was applied after this categorization, and the single parasite ASV which was detected in both samples and the extraction and sequencing controls (a fungus) was excluded from further analyses.

Unique 18 S sequences do not necessarily come from distinct species or even individuals, as each cell contains hundreds of copies of the 18 S gene, with subtle variation sometimes present between copies. We used a paired phylogeny and read abundance corelation approach to detect such sequences and remove false inflation of the parasite diversity. ASVs which are both closely related phylogenetically and strongly correlated in read abundance across samples are most parsimoniously explained as deriving from a single parasite species. Parasite ASV sequences were aligned using ClustalW in Mega4 with default parameters (Higgins and Sharp 1988; Tamura et al. 2007). A neighbour-joining tree was then constructed from this alignment, again using default Mega4 parameters for non-protein coding sequence data (Saitou and Nei 1987). Sequence read abundance correlations were calculated across all samples from normalized read count data, including each parasite ASV, and the categorical sums for all krill ASVs and all prey ASVs in MatLab. Correlations with a strength greater than 0.2 were visualized in a circle plot using the circularGraph package in MatLab (Kassebaum 2024).

Interactions between parasites and growth rate were explored using two approaches. For epibiotic ciliates (ciliate groups C, D, and E), a simple linear correlation approach was applied as these parasites were abundant at only one of the sampling locations. Linear correlations were calculated from percent sequence reads, using the polyfit function in MatLab. As apicomplexa parasites were present across all sampling locations, a generalized additive modelling (GAM) approach was taken with these, to account for variation between stations in the environmental parameters (temperature and chlorophyll) known to be related to growth in *E. superba* (Tarling et al. 2006). GAM models were built in MatLab using the fitrgam function, starting with a model including only the two environmental factors. A suite of models were then built expanding on this base model, each testing a different metric for quantifying apicomplexa infection intensity, including separating group A from the remaining groups (% apicomplexa reads on bodies, % apicomplexa reads on moults, % apicomplexa group A reads on bodies, % apicomplexa group A reads on moults, % apicomplexa non-A reads on bodies, % apicomplexa non-A reads on moults) (Figure S1). Lastly, a final suite of models incorporating environmental factors and apicomplexa load was constructed allowing for interaction between any of the three predictor variables. Hyperparameters were optimized for the maximum number of splits (1–20), maximum number of trees (1–500) and initial learning rate for predictors (0–0.25) using bayesopt. Each model was run both as a single run to calculate the partial dependencies of each of the predictor variables, and using 20 kfold partitions to calculate the kfold loss as a measure of the error in each model. The model which explained the largest proportion of the observed variation (Temperature, Chlorophyll, % apicomplexa reads on moults) was then applied to further analyses.

The potential population level impacts of observed parasites on krill were estimated by scaling up the calculated impacts of parasites on growth. We applied the simplifying assumptions that all krill are 41 mm in length (the population average in Tarling et al. 2016), and applied Morris et al.’s (1988) length (mm) to wet weight (grams) relationship of $\:Wet\:weight=0.00000339*{Length}^{3.23}$. We used the total global *E. superba* biomass of 379 million tons from Atkinson et al. (2009), which is within the range of estimates summarized in Siegel and Watkins (2016). We assumed a uniform intermoult period of 18 days, based on the average from Tarling et al. (2006), and the conservative assumption that krill only grow during peak summer of November through February. For both apicomplexa and ciliates, we calculated the expected change in krill biomass over a year in the absence of any parasites (e.g. applying the IGR values predicted by the GAM and linear fit for a parasite level of zero), and the expected change in biomass given the observed level of parasites. For ciliates, we applied the mean ciliate abundance across all stations to the linear fit, for apicomplexa we applied the step change in growth rate to the proportion of observed krill with parasite loads above the 38% threshold. The difference between these two values (biomass created without parasites– biomass created with parasites) was interpreted as the impact of parasites on krill biomass production at an annual scale.

## Results

Following quality control, a total of 29,806,668 sequence reads were included in the analyses across 198 samples (100 krill bodies and 98 moults as 2 moults failed to produce usable data). The number of sequence reads per sample was similar across both krill and moults, though with higher variability in moults (bodies mean 157,373 stdev 76,920; moults mean 140,352 stdev 117, 832). Of these sequence reads 57.7% (17,185,446 reads) were from krill itself, 7.8% (2,318,566) were from likely prey organisms, and the remaining 34.6% (10,302,656) were from parasitic organisms. The proportion of parasite reads was highly variable between samples, with samples of krill bodies containing between 0 and 96.8% parasite sequences (mean 13.8%), and moult samples containing between 0 and 99.4% (mean 49.9%).

**Fig. 1:**
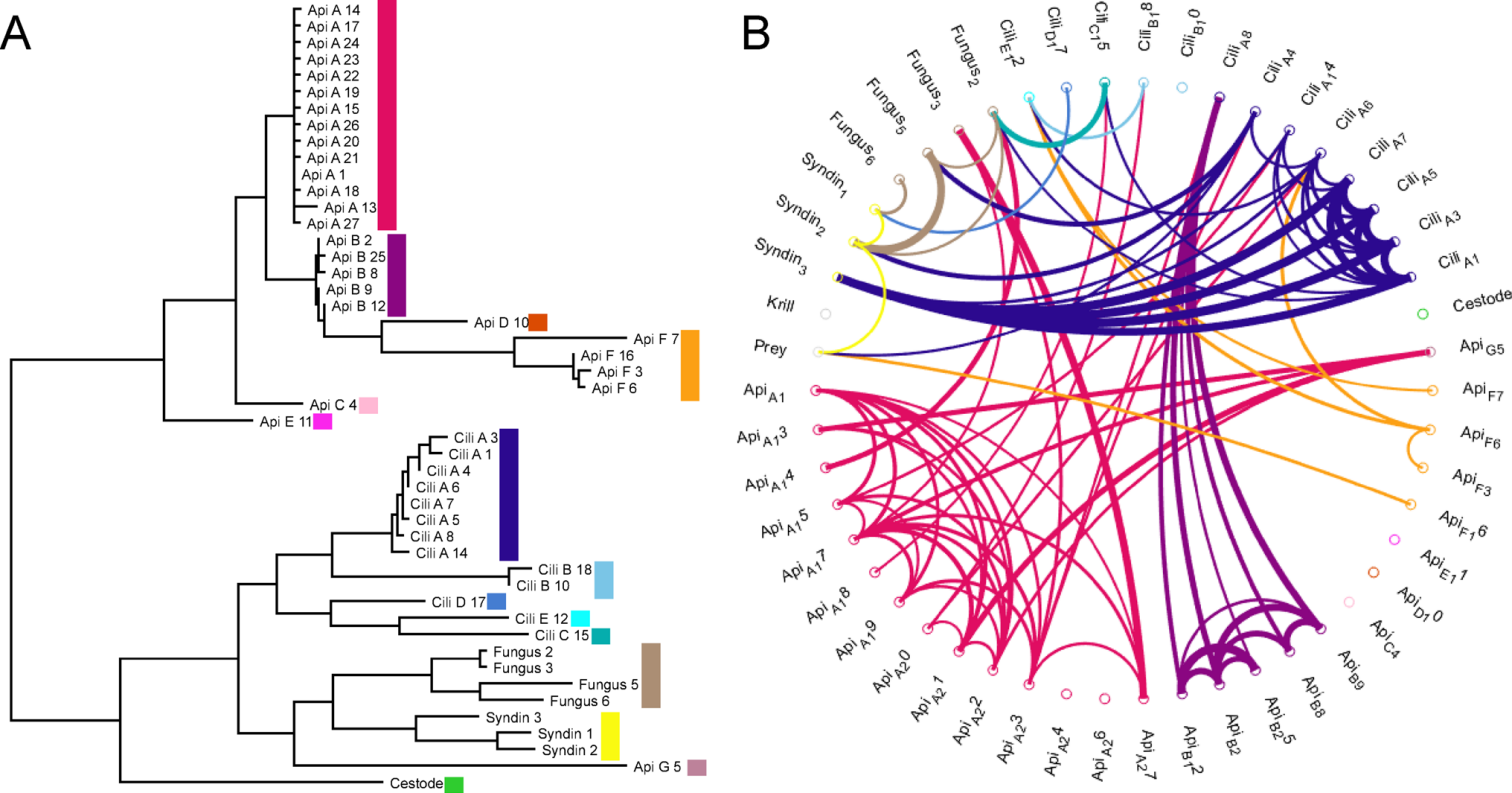
Phylogenetic and correlational analyses for clustering all unique parasite sequences. (**A**) Phylogenetic tree, total horizontal distance between sequences indicates relatedness (**B**) Correlation analyses, line thickness indicates strength of correlation (thinnest line = 0.20, thickest line = 0.99, correlations < 0.2 not shown). Both panels are coloured to indicate the parasite sequence groups which are clustered in the remaining analyses. Api = Apicomplexa, Cili = Ciliate, Syndin = Syndiniales

These 10 million sequence reads from parasitic organisms came from 48 unique sequences, or ASVs. Parasitic ASVs showed clear clustering both in phylogenetic analyses of the sequences themselves, and in cross-correlation analyses of the abundance of sequence reads across samples, and this clustering was consistent between the two approaches (Fig. 1). Based on this paired clustering approach, parasite ASVs were collapsed down to 15 distinct types for further analyses: seven apicomplexa, five ciliates, one fungus group, one *Parorchites zederi* cestode, and one syndiniales group (Fig. 1). Correlational analyses are less informative for the rarer groups, and this paired clustering approach may underestimate the diversity in the two least abundant groups (fungus & syndiniales), but as these groups are also least likely to be ecologically significant due to their relative rarity, this is a minor consideration.

**Fig. 2:**
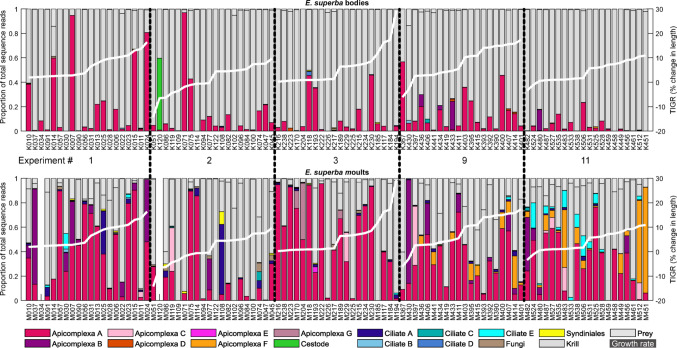
Distribution of parasite sequence reads across samples of krill bodies and moults. Samples are arranged by sampling location, and ordered within location by increasing growth rate (white line)

Apicomplexa were the most common parasite sequence group observed with 9,570,883 sequences (92.9% of all parasite reads). These gut parasites were also frequent across samples, with 45 of 100 krill bodies and 84 of 98 moults containing greater than 5% apicomplexa reads (of the total sample reads) (Fig. 2). The 27 apicomplexa ASVs clustered into 7 distinct groups (Fig. 1). All of these apicomplexa were most closely related to eugregarines, with groups A-F confidently placed within the Porosporoidea (Salomaki et al. 2021). Within this, groups A-E were most similar to sequences from various *Cephaloidophoria* spp., while group F was more closely associated with *Thirotia* spp. Apicomplexa group G, composed of a single ASV, was less consistently placed in phylogenetic analyses, but BLAST searches against GenBank reliably associated it with the Gregarinidae Apicomplexa. None of the Apicomplexa sequences recovered were 100% identical to reference sequences of taxonomically described Apicomplexa, so while the distinctness of the ASV groups and the overall association with eugregarines are clear, exact taxonomic identities are less certain. The most abundant Apicomplexa sequence, within group A, was 100% identical to existing sequences obtained from bulk gut samples of both *E. superba* and the ascidian tunicate *Ciona intestinalis*, so although the precise taxonomy remains uncertain, this organism has previously been recorded in similar hosts (Martin et al. 2006; Zhao 2015).

Ciliates were the second most abundant group of parasite sequences, with 518,100 sequences in 13 ASVs grouping into 5 clusters (Fig. 1). These sequences were split between internally infecting ciliates related to *Collinia* and *Pseudocollinia* spp. (groups A & B, 235,023 total reads, 2.3% of all parasite reads) and externally infesting ciliates related to *Ephelota* spp. (groups C, D, & E, 283,077 total reads, 2.7% of all parasite reads). The most abundant sequence within group A was 100% identical to sequences previously recovered from *E. superba* in the West Antarctic Peninsula, with a closest taxonomically described match at 98.7% similarity to *Lynnia grapsolytica* parasites from striped shore crabs (Cleary et al. 2019; Metz and Hechinger 2021). Ciliate group E sequences, the most abundant of the epibiotic ciliates observed, were 99.6% identical to references for *Ephelota plana* ciliates (Sato et al. 2015). Neither endoparasitc nor epibiotic ciliates exceeded 5% in any of the krill body samples. Endoparasitic ciliates exceeded 5% of the reads on 5 moult samples, all from experiments 1&2, while epibiotic ciliates exceed 5% of the reads on 9 moults samples, 8 of which were from experiment 11. Some patterns were observed between stations in Apicomplexa F and ciliate E, both of which were more abundant at the warmer and more oceanic stations (Figure S2).

Cestode sequences were the third most abundant group of parasite sequences, with 123,687 identical sequences, all found within a single individual krill body (Fig. 1). This cestode sequence was 100% identical to *Parorchites zederi* cestode isolated from Emperor penguins in East Antarctica (Kleinertz et al. 2014), and 99.7% identical to reference sequences of *P. zederi* from Chinstrap Penguins in the South Shetlands (Vidal et al. 2012).

Fungi and syndiniales were both relatively rare, compared to other parasite groups, with a total of 55,683 fungal sequences and 34,303 sequences from syndiniales (Fig. 1). Reference sequences for these two groups are limited, and 18 S is not particularly variable among the fungi, both of which limit the taxonomic resolution available for these ASVs (Hibbett et al. 2011).

**Fig. 3:**
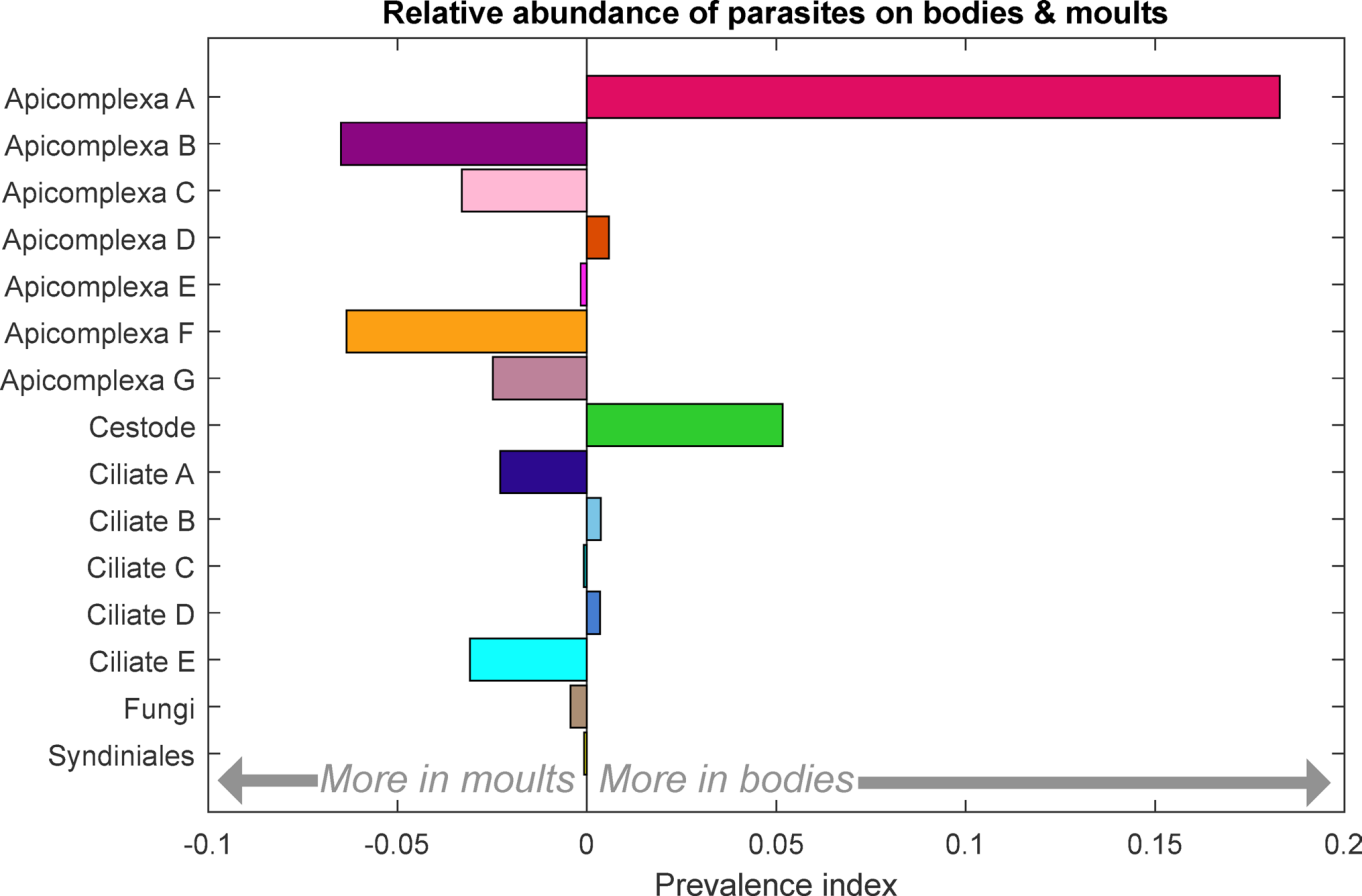
Relative abundance of each parasite in bodies vs. moults. The prevalence index is calculated as [(body parasite X/body total parasite) - (moult parasite X/moult total parasite)], to account for the proportional nature of the sequencing data and the differences between the sample types

Direct comparisons between the parasite proportions observed in bodies and those found on moults are complicated by the proportional nature of metabarcode sequencing (Deagle et al. 2019). A relatively lower proportion of parasites in bodies may reflect a lower parasite abundance on bodies, or a greater abundance of krill’s own DNA in the body tissues. Some patterns though are noticeable when looking at the read proportions (Fig. 2). These were explored further using a relative prevalence index calculated for each parasite type (X) as [(body parasite X/body total parasite) - (moult parasite X/moult total parasite)] (Fig. 3). Apicomplexa group A and cestode were more prevalent in body samples, Apicomplexa groups B, C, F & G and ciliates A & E were more prevalent on moults, while the remaining seven parasite groups showed no clear trend.

**Fig. 4:**
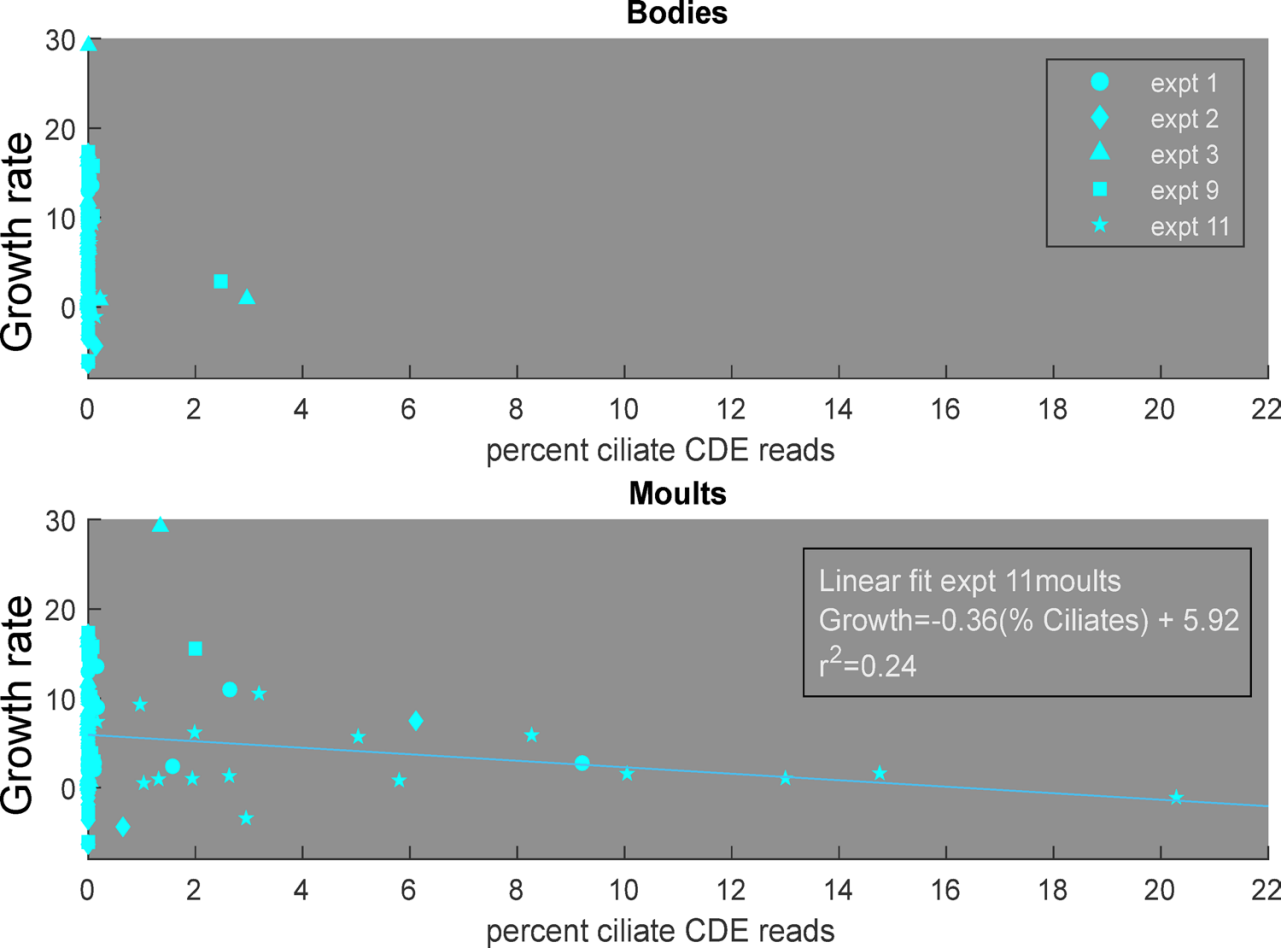
Correlations between the percent of sequences from epibiotic ciliate groups with krill growth rate

**Fig. 5:**
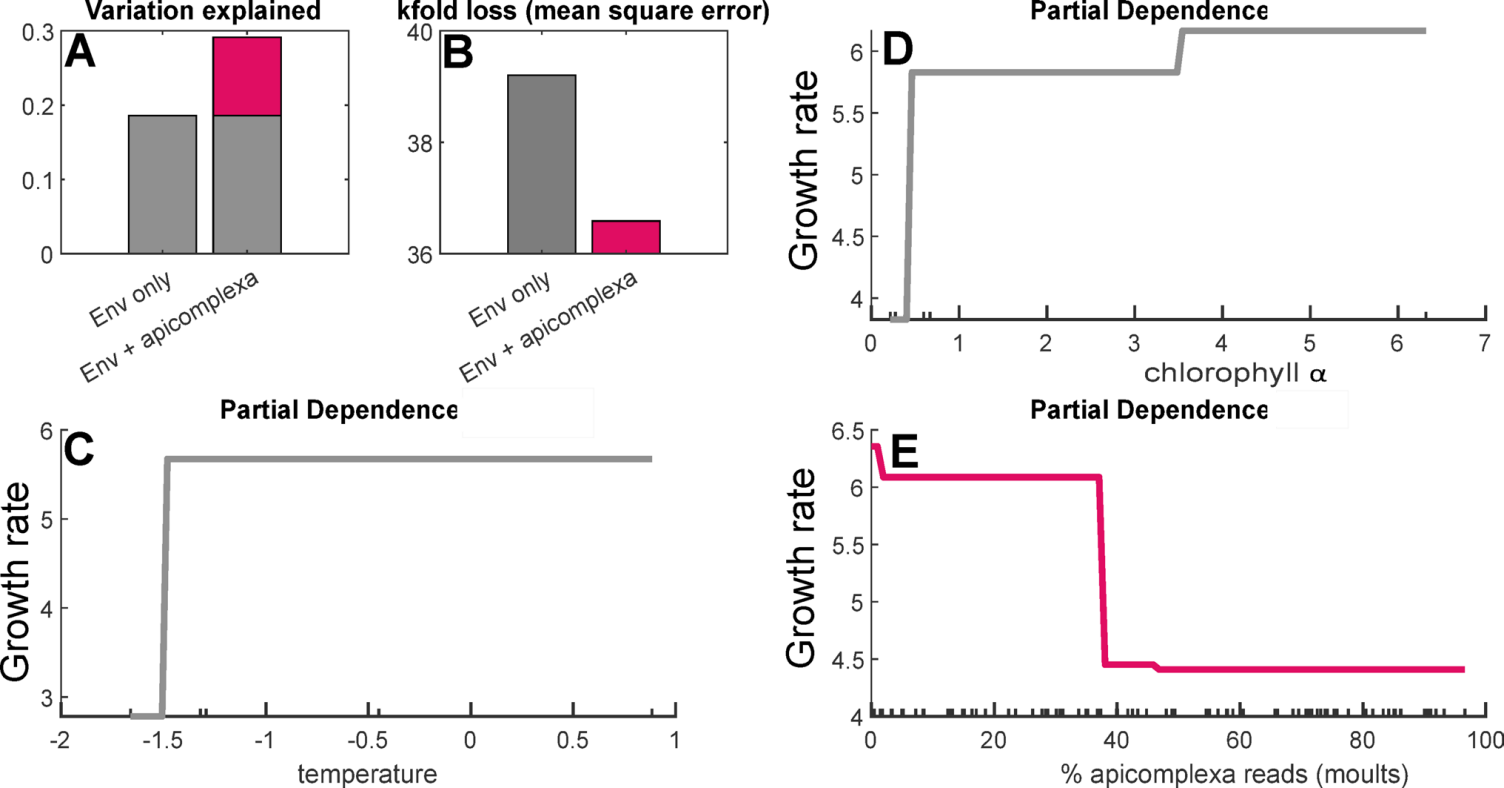
Generalized additive model testing the role of Apicomplexa in krill growth. (**A**) Variance explained by models including only environmental data (chlorophyll & temperature), or with the addition of Apicomplexa load as a variable, (**B**) Mean square error for these same two models, (**C**) Partial Dependence of growth on temperature, (**D**) Partial dependence of growth on chlorophyll α, (**E**) Partial dependence of growth on the load of Apicomplexa (% sequence reads on moults)

Growth rates decreased with increasing proportions of both epibiotic ciliates and Apicomplexa. The relationship between epibiotic ciliate proportion (as recorded by moults) and growth was estimated with a simple linear fit for the single incubation experiment where these ciliates were common. This fit, $\:Growth\:Rate=-0.36*\left(\%\:Ciliate\:Reads\right)+5.92$ explained over 20% of the variation in growth (r^2^ = 0.24) (Fig. 4). The relationship between Apicomplexa parasites and growth was estimated using GAMs to account for the variation in growth caused by the environment. A GAM predicting growth rate from only temperature and chlorophyll explained 18.6% of the variation; adding the percent Apicomplexa on moults increased the explanatory power to 29.1% (Fig. 5A). Other tested metrics for Apicomplexa infection intensity had less explanatory power. Including Apicomplexa on moults also decreased the mean square error (kfold loss) from 39.2 (environment alone) to 36.6 (environment + Apicomplexa) (Fig. 5B). Partial dependencies of the environmental predictor values fit expectations from literature, with growth increasing with increasing temperature and chlorophyll, over the ranges observed in this region. Partial dependence of growth rate on Apicomplexa showed decreasing growth rates with increasing parasite load, with a step change reduction at 38% Apicomplexa (Fig. 5E). No significant interactions (*p* < 0.5) were detected between Apicomplexa load, chlorophyll, and/or temperature.

Scaling the observed impact of parasites on krill growth, and the observed frequencies of parasites across the krill population, led to estimates that ciliates may reduce the biomass produced by 7.8%, while Apicomplexa may be responsible for a reduction of 14.9%. At an annual level, this would amount to 120.3 million tons of krill biomass effectively lost to parasites across the Southern Ocean.

## Discussion

### Parasite assemblage

The assemblage of parasites observed included both groups relatively commonly reported in *E. superba* (apicomplexa & ciliates), as well as less familiar groups (fungi & syndiniales), and parasites not previously known to infect this krill species (cestodes). These parasites belonged to 15 distinct groups. DNA sequence variation does not exactly correlate to traditional species delimitations, however the strong concordance between phylogenetic clustering and read abundance correlations provides confidence that these 15 parasite groups are distinct in biologically meaningful ways.

The most abundant parasite sequence group observed here, the Apicomplexa, are known gut parasites of *E. superba*. The porosporoidea Apicomplexa infect a broad range of marine crustaceans, and are well adapted metabolically to the hypoxic conditions of krill’s digestive tract (Salomaki et al. 2021). Our Apicomplexa groups A-E are closely affiliated with sequences from euphausiids, while group F shows similarities with the *Thiriotia* spp. gregarines, a group of Apicomplexa known from copepods and amphipods, but not previously noted in *E. superba* (Wakeman et al. 2018, 2021). Apicomplexa group F showed a clear spatial pattern, with higher proportions in the warmest and most oceanic stations, which may reflect a more cosmopolitan lifestyle, as compared to those Apicomplexa more closely associated with Antarctic euphausiids (Figure S2). Previous studies have also noted a higher prevalence of some Apicomplexa at sampling stations further from the coast, which may reflect similar drivers of Apicomplexa distributions (Xiong et al. 2024). Apicomplexa have been reported on multiple occasions in *E. superba*, and can be frequent across individuals, with estimates from various geographic areas ranging from 87 to 100%, which is consistent with our observations of 86% of moults showing substantial proportions of Apicomplexa sequence reads (Takahashi et al. 2008, 2009; Gómez-Gutiérrez and Morales-Ávila 2016; Avdeev and Avdeeva 1989).

The parasitic ciliates observed here have also been reported multiple times infesting *E. superba* and other euphausiids. *Ephelota* spp. ciliates have been observed both as active feeding stages, mainly attached to krill swimming legs and other areas of the body with high water flow rates, and as resting cysts, often attached to less exposed parts of krill bodies (Rakusa-Suszczewski and Nemoto 1989; Stankovic et al. 2002). These epibiotic ciliates can be common, Rakusa-Suszczewski and Nemoto (1989) observed infection rates of 35–72% of individuals (mean 55%) in east Antarctica, with highest infection rates among juveniles, while Stankovic et al. (2002) found an average infection rate by of 80% in samples from King George Island, Elephant Island, and the Bransfield Straight, although with a notable absence of *Ephelota* spp. on krill from the South Orkneys. Endoparasitic ciliates related to *Collinia* and *Pseudocollinia* spp. were less striking in the results, which is interesting as similar genetic analyses in the West Antarctic Peninsula found these to be the most common parasites in *E. superba* (Cleary et al. 2019). Applying the infection threshold used in the Peninsula study (> 50 reads/krill) though shows similar infection rates (this study 36%, Cleary et al. 2019 12%), suggesting the apparent difference in importance may reflect the variation in abundances of other parasite groups or an artefact of sample processing differences.

In contrast to the well-known Apicomplexa and ciliate parasites of Antarctic krill, cestodes of any kind have not previously been reported infecting *E. superba*, although they are known parasites of other euphausiid species (Gómez-Gutiérrez and Morales-Ávila 2016). The cestode identified here, *Parorchites zederi*, is known to infect a variety of Antarctic seabirds, and has also been identified in the krill-eating crab and leopard seals (Kleinertz et al. 2014). This has led to the speculation that *E. superba* are the likely intermediate hosts for this metazoan parasite, but ours is the first report of direct observation of *P. zederi* within *E. superba*.

The parasites identified here include most of the groups which have previously been reported from *E. superba*, though there are a few exceptions. These absences include *Foetteringidae* ciliates, Ellobiopsidae, and nematodes (Gómez-Gutiérrez and Morales-Ávila 2016). Ellobiopsidae and nematodes have been detected in previous studies applying these same genetic methods in *E. superba*, suggesting these absences are unlikely to be methodological artifacts (Cleary et al. 2019, 2024). However, neither of these parasites has previously been observed to be common, so while these absences may reflect differences in parasite prevalence across regions, larger sample sizes would be necessary to reach conclusions.

### The interaction of moulting and parasites

Euphausiids moult continuously throughout their lives, unlike most crustaceans which cease moulting once they reach a terminal adult size (Buchholz 1991). Moulting also occurs year-round, including during the winter period when classical drivers of moulting, including growth and reproduction are minimal or absent (Buckholz et al. 2006). Moulting is an energetically costly processes, and it remains unclear what drives this process to persist in adulthood and year-round for euphausiids (Buchholz et al. 2006). It has been suggested that ridding themselves of parasites may be one such driver (Buchholz et al.; Tarling and Cuzin-Roudy 2008). Krill moults replace both the exterior of the krill, and parts of the gut lining (Ikeda et al. 1984), thus potentially removing both epibionts and some gut parasites. Infestation intensity of epibiotic ciliates, including *Ephelota* spp., is typically highest in pre-moult krill, and lowest in post-moult individuals, indicating ciliates accumulate over the moult cycle (Endo et al. 2017; Tarling and Cuzin-Roudy 2008). Apicomplexa have also been observed to shift their distributions within the gut to avoid being lost during moulting (Takahashi et al. 2003). Our results are consistent with ciliates and some Apicomplexa groups being shed during moulting, although the proportional nature of metabarcoding means this finding should be viewed with some caution, and further research could benefit from applying a parasite-specific quantitative PCR approach to answer this question directly. It is interesting to note that our Apicomplexa group A which is most closely related to those typically reported in *E. superba* shows relatively higher prevalence within bodies, while our Apicomplexa group F, which is more closely related to parasites typically reported in copepods & amphipods, shows higher prevalence on moults. This may suggest that Apicomplexa group A has evolved to withstand moulting events, potentially as a result of a persistent association with euphausiids.

### Impacts of parasites on growth

Growth rates decreased with increasing proportions of both Apicomplexa and epibiotic ciliates. Additionally, although sample sizes are too small for statistical significance it is of note that the single krill infected by a cestode had the second lowest growth rate of all individuals measured, and was indeed shrinking, rather than growing. There are three potential explanations for these patterns– parasites preferentially infect poor condition krill, heavily parasitized krill moult more frequently, or parasites reduce krill growth. There is little evidence to suggest protistan parasites are able to target individual hosts, and similarly little indication of krill being able to control moult timing, so we here focus on the last explanation. Similarly, while the spatial and temporal scale of our data are limited and do increase the uncertainty of our findings, the range of environmental conditions is not unusual for the Southern Ocean, and at a broad level they are likely to be indicative of processes occurring widely in this ocean basin.

The observation that krill with high parasite loads have lower growth rates makes mechanistic sense and is consistent with existing reports that parasites of krill and other crustacean zooplankton can have significant physiological impacts on their hosts (Avdeev and Vagin 1987; Skovgaard 2014). Apicomplexa infections in *E. superba* have been shown to cause damage to the microvilli in the gut, which is thought to reduce the efficiency with which the krill host is able to absorb nutrients from ingested food (Takahashi et al. 2009). As food availability is a known driver of growth rates in *E. superba*, it is logical that a parasite which reduces krill’s ability to absorb ingested food would lead to lower growth rates. Cestodes have never before been reported in *E. superba*, but in other crustacean hosts they cause a wide range of changes in physiology (oxygen metabolism, immune responses, etc.) and behaviour (activity levels, responses to stimuli), which could impact energy budgets (Sánchez et al. 2009; Franceschi et al. 2007). Less is known about the physiological impacts of epibiotic ciliates such as those found here, but it is suggested that heavy infestations may increase hydrodynamic drag (Gómez-Gutiérrez and Morales-Ávila 2016). If krill need to invest more energy in swimming to compensate for this increased drag, this would reduce their total available energy budget, likely leaving less resources available for somatic growth.

Scaling our observations up to the level of krill populations, we calculate that Apicomplexa and epibiotic ciliate infections, at the levels observed in East Antarctica, would be responsible for 14.7 and 7.8% reductions, respectively, in the total krill biomass created, and equivalent to a total global reduction of 120.3 million tons of krill biomass annually. This falls within the range of estimates of total annual consumption of krill by vertebrate predators of 90–387 million tons (Trathan and Hill 2016). While there remains much uncertainty, it is striking that parasites may potentially be responsible for as much reduction in krill biomass as the classical predators. Parasite-host interactions are dynamic, and may shift under changing environmental conditions, seasonally, spatially and in relation to long term trends such as climate change. The frequency of parasites and the magnitude of the observed impact of parasites on growth of *E. superba* both highlight the need to better understand the role of parasites in Southern Ocean ecosystems, in order to inform sustainable management of living resources in a changing environment.

## Electronic supplementary material

Below is the link to the electronic supplementary material.

Supplementary Material 1

## Data Availability

Raw sequence data is available from the National Center for Biotechnology Information (NCBI) Short Read Archive (SRA) under bioproject PRJNA1069006. Assembled sequences from each of the identified parasite ASVs are available in NCBI’s GenBank under accession numbers PQ268164-PQ268211 Bioinformatics processing code and MatLab code for statistical analyses, modelling, and visualisations are available in the supplementary material.
