# Supplementary material for: Antarctic Krill with parasites grow slower than uninfected peers
Source: Mar Biol. 2025 Jun 11;172(7):113. doi: 10.1007/s00227-025-04673-w (PMC12158828; doi:10.1007/s00227-025-04673-w)
Supplement: Supplementary file 1 — Supplementary Material 1 [file 227_2025_4673_MOESM1_ESM.pdf]

Supplemental material for:

<u>Antarctic krill with parasites grow slower than uninfected peers</u>

AC Cleary, S Kawaguchi, R King, JE Melvin, GA Tarling

Contents:

Figure S1 Apicomplexa variable selection for GAM models……………………………………………page 2

Figure S2 Correlation of Apicomplexa group F with temperature……………………………………page 3

Bioinformatic processing script (python/BASH)……………………………………………………………….page 4

Data processing and visualization script (MatLab)…………………………………………………………page 7

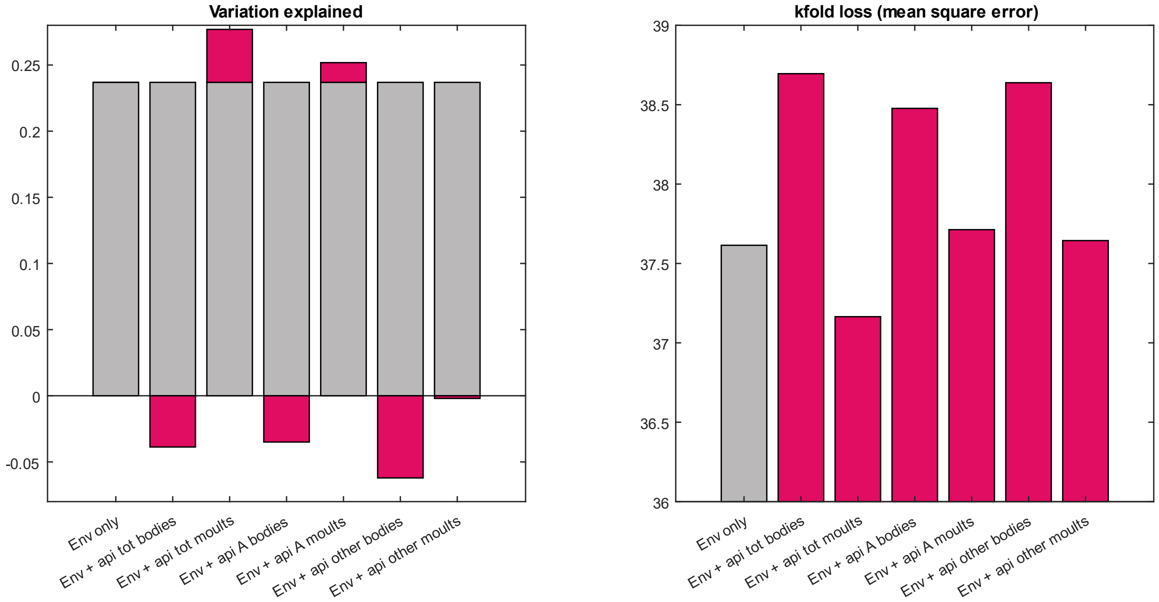

Figure S1: Comparison of metrics for modelling impacts of apicomplexa on krill growth. Total apicomplexa load on moults provides the greatest explanatory power and greatest reduction in mean square error.

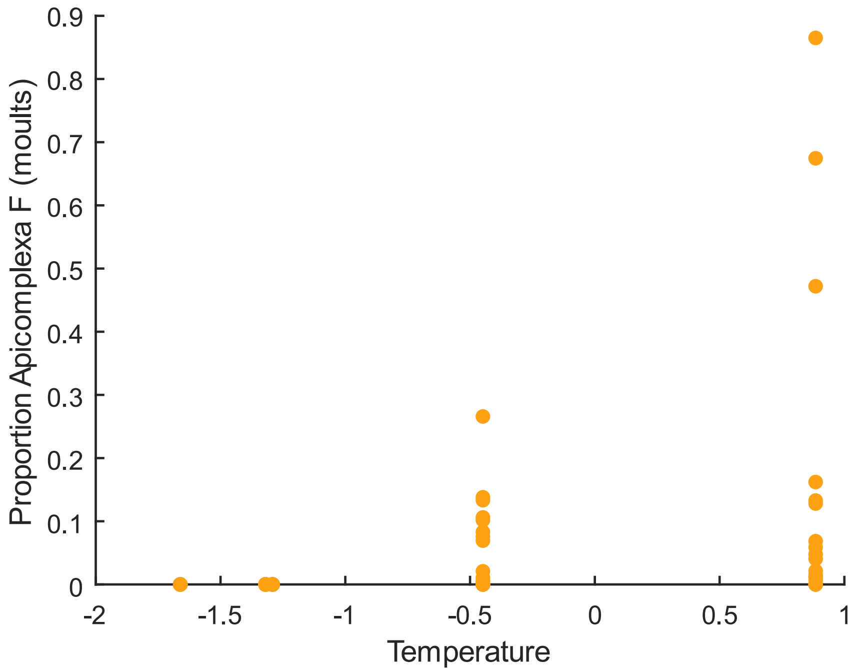

Figure S2: Apicomplexa F sequences on moults were only found in krill from the two warmest sampling locations, with the highest values found at the warmest station.

Bioinformatic processing script (python/BASH)

```
#!/bin/bash
#Job name:
#SBATCH --job-name=18S_processing
#
# Project:
#SBATCH --account=medium
#
# Wall clock limit:
#SBATCH --time=06:00:00
#
#Max memory usage per core (MB):
#SBATCH --mem-per-cpu=16G
#
#Number of cores:
#SBATCH --cpus-per-task=8
#
#Send the output to the qiime directory
#SBATCH -o /data/hpcdata/users/ANONYMYZED/run_files/Qiime_2024_%j.out
#SBATCH -D /data/hpcdata/users/ ANONYMYZED/
#
#SBATCH --partition=medium

# Enable software modules
source /etc/profile.d/modules.sh

## Set up job environment:
#source /cluster/bin/jobsetup
module purge   # clear any inherited modules
set -o errexit # exit on errors
set -o nounset # Treat unset variables as errors
#mkdir -p /data/hpcdata/users/ ANONYMYZED /tmp/
export TMPDIR=/data/hpcdata/users/ ANONYMYZED /tmp/
export MPLCONFIGDIR=/data/hpcdata/users/ANONYMYZED /tmp-mplconfigdir
export HOME=/data/hpcdata/users/ ANONYMYZED
mkdir -p $MPLCONFIGDIR $TMPDIR

module load hpc/qiime2/2022.2
module list

echo "The script is running"

cd /data/hpcdata/users/ ANONYMYZED/WP2_analysis_files/

echo "Now I am in the analysis folder, this folder contains:"

ls
```

##Copy the data into one oerall file folder ready for qiime analysis
mkdir /data/hpcdata/users/ANONYMYZED/analysis_files/raw_reads/
mkdir ../../data/hpcdata/users/ANONYMYZED/analysis_files/2024/
cp -t /data/hpcdata/users/ANONYMYZED/analysis_files/raw_reads/
/data/hpcdata/users/ANONYMYZED/illumina_reupload/*/*/*.fastq.gz

Files delivered as demultiplexed. They import with the Casava-18 format import tool.
qiime tools import \
  --type 'SampleData[PairedEndSequencesWithQuality]' \
  --input-path ../../data/hpcdata/users/ANONYMYZED/analysis_files/raw_reads \
  --input-format CasavaOneEightSingleLanePerSampleDirFmt \
  --output-path ../../data/hpcdata/users/ANONYMYZED/analysis_files/All_reads_imported.qza

#Trim the reads to remove forward and reverse primers, from both forward and reverse reads. This is completed in two steps in order to allow greater leniency with the primer at the end of each read and avoid biasing against longer amplicons.
#18S reads primers are (Forward – 5’ CGG CTY AAT TYG AYT CAA CRC 3’, Reverse – 5’ GGG CAT CAC RGA CCT G 3’
qiime cutadapt trim-paired \
  --i-demultiplexed-sequences All_reads_imported.qza \
  --p-front-f CGGCTYAATTYGAYTCAACRC \
  --p-front-r GGGCATCACRGACCTG \
  --p-error-rate 0.1 \
  --p-match-adapter-wildcards \
  --p-discard-untrimmed \
  --o-trimmed-sequences 2024/All_18S_forward_trimmed_sequences.qza \
  --verbose | tee 2024/All_18S_forward_trimming_log.txt

qiime cutadapt trim-paired \
  --i-demultiplexed-sequences 2024/All_18S_forward_trimmed_sequences.qza \
  --p-adapter-f CAGGTCYGTGATGCCC \
  --p-adapter-r GYGTTGARTCRAATTRAGCCG \
  --p-match-adapter-wildcards \
  --p-error-rate 0.1 \
  --o-trimmed-sequences 2024/All_18S_fully_trimmed_sequences.qza \
  --verbose | tee 2024/All_18S_reverse_trimming_log.tx

#Denoise the reads, which joins the two ends and corrects errors or removes sequences which contain too many errors
#18S denoising expected total amplicon size is around 250, reads are 300 bp
qiime dada2 denoise-paired \
   --i-demultiplexed-seqs 2024/All_18S_fully_trimmed_sequences.qza \
   --p-trunc-len-f 185 \
   --p-trunc-len-r 180 \
   --p-trunc-q 2 \
   --p-max-ee-f 3 \
   --p-max-ee-r 3 \

```
  --p-min-fold-parent-over-abundance 1 \
  --p-pooling-method pseudo \
  --o-table 2024/All_18S_ASV_count_table_185_180.qza \
  --o-representative-sequences 2024/All_18S_rep_set_185_180.qza \
  --o-denoising-stats 2024/All_18S_ASV_dada2denoising_log_185_180.qza \
  --p-n-threads 0 \
  --verbose

#Assign 18S taxonomy using a machine learning classifier trained specifically on the Gast amplicons
qiime feature-classifier classify-sklearn \
  --i-reads 2024/All_18S_rep_set_185_180.qza \
  --i-classifier ../Reference_sequences/silva-138-99-amplicon-classifier.qza \
  --o-classification 2024/All_18S_taxonomy.qza \
  --verbose

##Convert the ASV table to a format that will open in Excell/Notepad++
unzip ANONYMYZED/analysis_files/All_18S_ASV_count_table_2.qza -d ANONYMYZED/analysis_files/
##manually re-name folder to Complete_ASV_count_table_inflated (unzipping gives it a difficult to predict name, hence the manual approach)
biom convert -i
/data/hpcdata/users/ANONYMYZED/analysis_files/All_18S_ASV_count_table_unzipped/data/feature-table.biom \
  -o /data/hpcdata/users/ANONYMYZED/analysis_files/All_18S_ASV_table.txt \
  --to-tsv
```

```
%This MatLab script provides various analyses and visualizations of the ASV count data (after quality control and taxonomy filtering

%ASV_table is copied in from excel, rows are ASVs, columns are
%individuals, final two rows are krill and prey

%The ASVs need to be normalized to account for variation in total sequence
%read depth per sample.
%exclude blanks and the two moult samples with no reads, re-order ciliate
%ASVs so that the highly correlated ones are next to each other for easy
%visualization.
ASV_table=ASV_table_May_28([1:29 31 33 35 34 30 32 36:end],[3:116 118:142 144:end]);

sample_sums=sum(ASV_table,1);

ASVs_normalized=ASV_table./sample_sums;

%The second step is to go from ASV table to a correlation matrix. This
%requires data to be in the format of rows as observations & columns as
%variables (so, invert the original)

ASVs_for_correlation=ASVs_normalized';

%calculate correlations
ASV_corr = corrcoef(ASVs_for_correlation);

%%
%Now make a heatmap
ASV_names_ord=ASV_names([1:29 31 33 35 34 30 32 36:end]);
figure;heatmap(ASV_names_ord, ASV_names_ord, ASV_corr, 'Colormap', jet)

%set colours to be consistent across all figures
Circle_colours_grouped=zeros(50,3);
Circle_colours_grouped(1:14,1:3)=repmat(Colour_api_A,14,1);
Circle_colours_grouped(15:19,1:3)=repmat(Colour_api_B,5,1);
Circle_colours_grouped(20,1:3)=Colour_api_C;
Circle_colours_grouped(21,1:3)=Colour_api_D;
Circle_colours_grouped(22,1:3)=Colour_api_E;
Circle_colours_grouped(23:26,1:3)=repmat(Colour_api_F,4,1);
Circle_colours_grouped(27,1:3)=Colour_api_G;
Circle_colours_grouped(28,1:3)=Colour_cestode;
Circle_colours_grouped(29:36,1:3)=repmat(Colour_cili_A,8,1);
Circle_colours_grouped(37:38,1:3)=repmat(Colour_cili_B,2,1);
Circle_colours_grouped(39,1:3)=Colour_cili_C;
Circle_colours_grouped(40,1:3)=Colour_cili_D;
Circle_colours_grouped(41,1:3)=Colour_cili_E;
Circle_colours_grouped(42:45,1:3)=repmat(Colour_fungi,4,1);
Circle_colours_grouped(46:48,1:3)=repmat(Colour_synd,3,1);
```

```
Circle_colours_grouped(49,1:3)=Colour_krill;
Circle_colours_grouped(50,1:3)=Colour_prey;

%And now make a circle plot
ASV_labels_ord=cellstr(ASV_names_ord);
ASVs_for_circle=ASV_corr.*(ASV_corr>0.2)*10;
figure;circularGraph(ASVs_for_circle,'Label',ASV_labels_ord,'Colormap',Circle_colours_grouped)

%% Now to make a bar plot
%because plotting every single ASV makes it unreadable, combining all
%within letter groups for apicomplexa and ciliates

%first consolidate categories
ASV_consol=zeros(17,202);
%apicomplexa
ASV_consol(1,:)=sum(ASV_table_May_28(1:14,:),1)./sum(ASV_table_May_28,1);
ASV_consol(2,:)=sum(ASV_table_May_28(15:19,:),1)./sum(ASV_table_May_28,1);
ASV_consol(3,:)=ASV_table_May_28(20,:)./sum(ASV_table_May_28,1);
ASV_consol(4,:)=ASV_table_May_28(21,:)./sum(ASV_table_May_28,1);
ASV_consol(5,:)=ASV_table_May_28(22,:)./sum(ASV_table_May_28,1);
ASV_consol(6,:)=sum(ASV_table_May_28(23:26,:),1)./sum(ASV_table_May_28,1);
ASV_consol(7,:)=ASV_table_May_28(27,:)./sum(ASV_table_May_28,1);
%cestode
ASV_consol(8,:)=ASV_table_May_28(28,:)./sum(ASV_table_May_28,1);
%ciliates
ASV_consol(9,:)=sum(ASV_table_May_28(29:36,:),1)./sum(ASV_table_May_28,1);
ASV_consol(10,:)=sum(ASV_table_May_28(37:38,:),1)./sum(ASV_table_May_28,1);
ASV_consol(11,:)=ASV_table_May_28(39,:)./sum(ASV_table_May_28,1);
ASV_consol(12,:)=ASV_table_May_28(40,:)./sum(ASV_table_May_28,1);
ASV_consol(13,:)=ASV_table_May_28(41,:)./sum(ASV_table_May_28,1);
%fungus
ASV_consol(14,:)=sum(ASV_table_May_28(42:45,:),1)./sum(ASV_table_May_28,1);
%syndiniales
ASV_consol(15,:)=sum(ASV_table_May_28(46:48,:),1)./sum(ASV_table_May_28,1);
%krill
ASV_consol(16,:)=ASV_table_May_28(49,:)./sum(ASV_table_May_28,1);
%prey
ASV_consol(17,:)=ASV_table_May_28(50,:)./sum(ASV_table_May_28,1);

%Now re-order so they make visual sense (remove samples with no data and
%order by station then growth rate)
ASV_for_bars=ASV_consol(:,[Plotting_order]);
TIGR_for_bars=Metadata_May_28([Plotting_order],8);
Sample_names_for_bars=Sample_names(Plotting_order);
ASV_names_for_bars= ["Apicomplexa A" "Apicomplexa B" "Apicomplexa C" "Apicomplexa D"
"Apicomplexa E" "Apicomplexa F" "Apicomplexa G" "Cestode" "Ciliate A" "Ciliate B" "Ciliate C"
"Ciliate D" "Ciliate E" "Fungi" "Syndiniales" "Krill" "Prey"];
```

```
figure;
sgtitle('Parasite Sequence Reads')
subplot(2,1,1);
hold on
%plot proportion of each ASV as stacked bars
yyaxis left
bars = bar(ASV_for_bars(:,1:100)','stacked', 'FaceColor','flat');
title('Bodies')
bars(1).CData = Colour_api_A;
bars(2).CData = Colour_api_B;
bars(3).CData = Colour_api_C;
bars(4).CData = Colour_api_D;
bars(5).CData = Colour_api_E;
bars(6).CData = Colour_api_F;
bars(7).CData = Colour_api_G;
bars(8).CData = Colour_cestode;
bars(9).CData = Colour_cili_A;
bars(10).CData = Colour_cili_B;
bars(11).CData = Colour_cili_C;
bars(12).CData = Colour_cili_D;
bars(13).CData = Colour_cili_E;
bars(14).CData = Colour_fungi;
bars(15).CData = Colour_synd;
bars(16).CData = Colour_krill;
bars(17).CData = Colour_prey;
xticks(1:100);
xticklabels(Sample_names_for_bars(1:100));
xtickangle(90);
ylim([0,1])
ylabel("% total sequence reads")
set(gca, 'XColor','k', 'YColor','k','FontSize', 10)
%legend(ASV_names_for_bars,'Location','southoutside','NumColumns',8)
%plot TIGR or other variable of interest as a line over the top
yyaxis right
p1 = plot(1:20,TIGR_for_bars(1:20),'w','LineWidth',3,'LineStyle','-','Marker','none');
p2 = plot(21:40,TIGR_for_bars(21:40),'w','LineWidth',3,'LineStyle','-','Marker','none');
p3 = plot(41:60,TIGR_for_bars(41:60),'w','LineWidth',3,'LineStyle','-','Marker','none');
p4 = plot(61:80,TIGR_for_bars(61:80),'w','LineWidth',3,'LineStyle','-','Marker','none');
p5 = plot(81:100,TIGR_for_bars(81:100),'w','LineWidth',3,'LineStyle','-','Marker','none');
ylabel("TIGR (% change in length)")
set(gca, 'XColor','k', 'YColor','k')
hold off
subplot(2,1,2);
hold on
yyaxis left
bars = bar(ASV_for_bars(:,101:200)','stacked', 'FaceColor','flat');
title('Moults')
bars(1).CData = Colour_api_A;
```

```
bars(2).CData = Colour_api_B;
bars(3).CData = Colour_api_C;
bars(4).CData = Colour_api_D;
bars(5).CData = Colour_api_E;
bars(6).CData = Colour_api_F;
bars(7).CData = Colour_api_G;
bars(8).CData = Colour_cestode;
bars(9).CData = Colour_cili_A;
bars(10).CData = Colour_cili_B;
bars(11).CData = Colour_cili_C;
bars(12).CData = Colour_cili_D;
bars(13).CData = Colour_cili_E;
bars(14).CData = Colour_fungi;
bars(15).CData = Colour_synd;
bars(16).CData = Colour_krill;
bars(17).CData = Colour_prey;
xticks(1:100);
xticklabels(Sample_names_for_bars(101:200));
xtickangle(90);
ylim([0,1])
ylabel("% total sequence reads")
set(gca, 'XColor','k', 'YColor','k')
%legend(ASV_names_for_bars,'Location','southoutside','NumColumns',10)
yyaxis right
p1 = plot(1:20,TIGR_for_bars(1:20),'w','LineWidth',3,'LineStyle','-','Marker','none');
p2 = plot(21:40,TIGR_for_bars(21:40),'w','LineWidth',3,'LineStyle','-','Marker','none');
p3 = plot(41:60,TIGR_for_bars(41:60),'w','LineWidth',3,'LineStyle','-','Marker','none');
p4 = plot(61:80,TIGR_for_bars(61:80),'w','LineWidth',3,'LineStyle','-','Marker','none');
p5 = plot(81:100,TIGR_for_bars(81:100),'w','LineWidth',3,'LineStyle','-','Marker','none');
ylabel("TIGR (% change in length)")
set(gca, 'XColor','k', 'YColor','k','FontSize', 10)
hold off


%% Now to make a bar plot which looks at the relative importance of bodies vs moults for each
parasite type, normalized by total parasites to account for the difference in krill tissue between
bodies and moults.

%first consolidate categories
ASV_parasites_B_M_ratio=zeros(15,1);
%apicomplexa
ASV_parasites_B_M_ratio(1)
=sum(sum(ASV_table_May_28(1:14,3:102),1),2)/sum(sum(ASV_table_May_28(1:48,3:102),1),2) -
sum(sum(ASV_table_May_28(1:14,103:202),1,"omitnan"),2,"omitnan")/sum(sum(ASV_table_May_2
8(1:48,103:202),1,"omitnan"),2,"omitnan");
ASV_parasites_B_M_ratio(2)
=sum(sum(ASV_table_May_28(15:19,3:102),1),2)/sum(sum(ASV_table_May_28(1:48,3:102),1),2) -
```

```
sum(sum(ASV_table_May_28(15:19,103:202),1,"omitnan"),2,"omitnan")/sum(sum(ASV_table_May_28(1:48,103:202),1,"omitnan"),2,"omitnan");
ASV_parasites_B_M_ratio(3)
=sum(ASV_table_May_28(20,3:102),2)/sum(sum(ASV_table_May_28(1:48,3:102),1),2) - sum(ASV_table_May_28(20,103:202),2,"omitnan")/sum(sum(ASV_table_May_28(1:48,103:202),1,"omitnan"),2,"omitnan");
ASV_parasites_B_M_ratio(4)
=sum(ASV_table_May_28(21,3:102),2)/sum(sum(ASV_table_May_28(1:48,3:102),1),2) - sum(ASV_table_May_28(21,103:202),2,"omitnan")/sum(sum(ASV_table_May_28(1:48,103:202),1,"omitnan"),2,"omitnan");
ASV_parasites_B_M_ratio(5)
=sum(ASV_table_May_28(22,3:102),2)/sum(sum(ASV_table_May_28(1:48,3:102),1),2) - sum(ASV_table_May_28(22,103:202),2,"omitnan")/sum(sum(ASV_table_May_28(1:48,103:202),1,"omitnan"),2,"omitnan");
ASV_parasites_B_M_ratio(6)
=sum(sum(ASV_table_May_28(23:26,3:102),1),2)/sum(sum(ASV_table_May_28(1:48,3:102),1),2) - sum(sum(ASV_table_May_28(23:26,103:202),1,"omitnan"),2,"omitnan")/sum(sum(ASV_table_May_28(1:48,103:202),1,"omitnan"),2,"omitnan");
ASV_parasites_B_M_ratio(7)
=sum(ASV_table_May_28(27,3:102),2)/sum(sum(ASV_table_May_28(1:48,3:102),1),2) - sum(ASV_table_May_28(27,103:202),2,"omitnan")/sum(sum(ASV_table_May_28(1:48,103:202),1,"omitnan"),2,"omitnan");
%cestode
ASV_parasites_B_M_ratio(8)
=sum(ASV_table_May_28(28,3:102),2)/sum(sum(ASV_table_May_28(1:48,3:102),1),2) - sum(ASV_table_May_28(28,103:202),2,"omitnan")/sum(sum(ASV_table_May_28(1:48,103:202),1,"omitnan"),2,"omitnan");
%ciliates
ASV_parasites_B_M_ratio(9)
=sum(sum(ASV_table_May_28(29:36,3:102),1),2)/sum(sum(ASV_table_May_28(1:48,3:102),1),2) - sum(sum(ASV_table_May_28(29:36,103:202),1,"omitnan"),2,"omitnan")/sum(sum(ASV_table_May_28(1:48,103:202),1,"omitnan"),2,"omitnan");
ASV_parasites_B_M_ratio(10)
=sum(sum(ASV_table_May_28(37:38,3:102),1),2)/sum(sum(ASV_table_May_28(1:48,3:102),1),2) - sum(sum(ASV_table_May_28(37:38,103:202),1,"omitnan"),2,"omitnan")/sum(sum(ASV_table_May_28(1:48,103:202),1,"omitnan"),2,"omitnan");
ASV_parasites_B_M_ratio(11)
=sum(ASV_table_May_28(39,3:102),2)/sum(sum(ASV_table_May_28(1:48,3:102),1),2) - sum(ASV_table_May_28(39,103:202),2,"omitnan")/sum(sum(ASV_table_May_28(1:48,103:202),1,"omitnan"),2,"omitnan");
ASV_parasites_B_M_ratio(12)
=sum(ASV_table_May_28(40,3:102),2)/sum(sum(ASV_table_May_28(1:48,3:102),1),2) - sum(ASV_table_May_28(40,103:202),2,"omitnan")/sum(sum(ASV_table_May_28(1:48,103:202),1,"omitnan"),2,"omitnan");
ASV_parasites_B_M_ratio(13)
=sum(ASV_table_May_28(41,3:102),2)/sum(sum(ASV_table_May_28(1:48,3:102),1),2) - sum(ASV_table_May_28(41,103:202),2,"omitnan")/sum(sum(ASV_table_May_28(1:48,103:202),1,"omitnan"),2,"omitnan");
```

```
%fungus
ASV_parasites_B_M_ratio(14)
=sum(sum(ASV_table_May_28(42:45,3:102),1),2)/sum(sum(ASV_table_May_28(1:48,3:102),1),2) -
sum(sum(ASV_table_May_28(42:45,103:202),1,"omitnan"),2,"omitnan")/sum(sum(ASV_table_May_
28(1:48,103:202),1,"omitnan"),2,"omitnan");
%syndiniales
ASV_parasites_B_M_ratio(15)
=sum(sum(ASV_table_May_28(46:48,3:102),1),2)/sum(sum(ASV_table_May_28(1:48,3:102),1),2) -
sum(sum(ASV_table_May_28(46:48,103:202),1,"omitnan"),2,"omitnan")/sum(sum(ASV_table_May_
28(1:48,103:202),1,"omitnan"),2,"omitnan");

figure;
bars = barh(ASV_parasites_B_M_ratio(1:15), 0.9, 'FaceColor','flat');
bars.CData(1,:) = Colour_api_A;
bars.CData(2,:) = Colour_api_B;
bars.CData(3,:) = Colour_api_C;
bars.CData(4,:) = Colour_api_D;
bars.CData(5,:) = Colour_api_E;
bars.CData(6,:) = Colour_api_F;
bars.CData(7,:) = Colour_api_G;
bars.CData(8,:) = Colour_cestode;
bars.CData(9,:) = Colour_cili_A;
bars.CData(10,:) = Colour_cili_B;
bars.CData(11,:) = Colour_cili_C;
bars.CData(12,:) = Colour_cili_D;
bars.CData(13,:) = Colour_cili_E;
bars.CData(14,:) = Colour_fungi;
bars.CData(15,:) = Colour_synd;
yticklabels(ASV_names_for_bars(1:15))
xlabel("Prevelance index")
ylabel("Parasite")
title('Relative abundance of parasites on bodies & moults')
set (gca,'YDir','reverse')


%% Switch to working with the GAM input file, Start with simple correlations

%Separate out tables for each expt and remove those indivs with no moult
%data
IGR_ex_1_GAM_table = Krill_for_GAM_refined(Krill_for_GAM_refined.experiment == 1 &
~isnan(Krill_for_GAM_refined.apicomplexa_moults), :);
IGR_ex_2_GAM_table = Krill_for_GAM_refined(Krill_for_GAM_refined.experiment == 2 &
~isnan(Krill_for_GAM_refined.apicomplexa_moults), :);
IGR_ex_3_GAM_table = Krill_for_GAM_refined(Krill_for_GAM_refined.experiment == 3 &
~isnan(Krill_for_GAM_refined.apicomplexa_moults), :);
IGR_ex_9_GAM_table = Krill_for_GAM_refined(Krill_for_GAM_refined.experiment == 9 &
~isnan(Krill_for_GAM_refined.apicomplexa_moults), :);
```

```
IGR_ex_11_GAM_table = Krill_for_GAM_refined(Krill_for_GAM_refined.experiment == 11 &
~isnan(Krill_for_GAM_refined.apicomplexa_moults), :);
IGR_all_ex_GAM_table =
Krill_for_GAM_refined(~isnan(Krill_for_GAM_refined.apicomplexa_moults), :);

%Make a two pannel scatter plot showing ciliate cde load on x axis and TIGR
%on Y-axis, including the line fit for station 11 where they are abundant.

IGR_ex_11_cil_cde_corrcoef=corrcoef(IGR_ex_11_GAM_table.("ciliates_cde_moults"),IGR_ex_11_G
AM_table.("TIGR"));
cili_CDE_r2=(IGR_ex_11_cil_cde_corrcoef(1,2))^2;
cili_cde_ex11_simple_line=polyfit(IGR_ex_11_GAM_table.("ciliates_cde_moults"),IGR_ex_11_GAM_
table.("TIGR"),1);
x = 0:25;
f = polyval(cili_cde_ex11_simple_line,x);

%bodies in first panel
figure;
hold on
sgtitle('TIGR as a function of ciliate CDE load  shapes indicate station')
subplot(2,1,1);
hold on
title('Bodies')
scatter(IGR_ex_1_GAM_table.("ciliates_cde_bodies"),IGR_ex_1_GAM_table.("TIGR"),25,Colour_cili_
E,'o','fill')
scatter(IGR_ex_2_GAM_table.("ciliates_cde_bodies"),IGR_ex_2_GAM_table.("TIGR"),25,Colour_cili_
E,'diamond','fill')
scatter(IGR_ex_3_GAM_table.("ciliates_cde_bodies"),IGR_ex_3_GAM_table.("TIGR"),25,Colour_cili_
E,'^','fill')
scatter(IGR_ex_9_GAM_table.("ciliates_cde_bodies"),IGR_ex_9_GAM_table.("TIGR"),25,Colour_cili_
E,'square','fill')
scatter(IGR_ex_11_GAM_table.("ciliates_cde_bodies"),IGR_ex_11_GAM_table.("TIGR"),30,Colour_cil
i_E,'pentagram','fill')
set(gca,'Color',Colour_env)
legend('expt 1','expt 2', 'expt 3', 'expt 9', 'expt
11','Location','northeast','Orientation','vertical','Textcolor',[0.9 0.9 0.9])
ylabel("TIGR")
xlabel("proportion ciliate CDE reads")
ylim([-8 30])
xlim([0 22])
subplot(2,1,2);
hold on
%moults in second panel
title('Moults')
scatter(IGR_ex_1_GAM_table.("ciliates_cde_moults"),IGR_ex_1_GAM_table.("TIGR"),25,Colour_cili_
E,'o','fill')
scatter(IGR_ex_2_GAM_table.("ciliates_cde_moults"),IGR_ex_2_GAM_table.("TIGR"),25,Colour_cili_
E,'diamond','fill')
```

```
scatter(IGR_ex_3_GAM_table.("ciliates_cde_moults"),IGR_ex_3_GAM_table.("TIGR"),25,Colour_cili_E,'^','fill')
scatter(IGR_ex_9_GAM_table.("ciliates_cde_moults"),IGR_ex_9_GAM_table.("TIGR"),25,Colour_cili_E,'square','fill')
scatter(IGR_ex_11_GAM_table.("ciliates_cde_moults"),IGR_ex_11_GAM_table.("TIGR"),30,Colour_cili_E,'pentagram','fill')
plot(x,f,'-')
ylim([-8 30])
xlim([0 22])
dim = [.6 .2 .2 .2];
str = {'Linear fit expt 11 moults', 'TIGR=-0.36(% Ciliates) + 5.92', 'r^2=0.24'};
annotation('textbox',dim,'String',str,'FitBoxToText','on','Color',[0.9 0.9 0.9]);
ylabel("TIGR")
xlabel("proportion ciliate CDE reads")
set(gca,'Color',Colour_env)
hold off

%% GAM modelling focusing on Apicomplexa because present across areas and at interpretable abundances.

% For all models optimize key hyperparameters.  Because cross
%validated GAM models do not output the partial
%dependence, run each model both with and without cross validation.

Krill_for_GAM=Krill_for_GAM_refined;
c = cvpartition(length(Krill_for_GAM.TIGR),'KFold',50);
maxNumSplits = optimizableVariable('maxNumSplits',[1,20],'Type','integer');
numTrees = optimizableVariable('numTrees',[1,500],'Type','integer');
learnpreds = optimizableVariable('learnpreds',[0,0.25],'Type','real');

%Auto option optimizes these: InitialLearnRateForPredictors, NumTreesPerPredictor, Interactions, InitialLearnRateForInteractions, and NumTreesPerInteraction.

minfun =
@(z)kfoldLoss(fitrgam(Krill_for_GAM,'TIGR',"PredictorNames",["chla","temperature"],'CVPartition',c,
...
    'MaxNumSplitsPerPredictor',z.maxNumSplits, ...
    'NumTreesPerPredictor',z.numTrees, 'InitialLearnRateForPredictors',z.learnpreds));
rng('default')
results = bayesopt(minfun,[maxNumSplits,numTrees,learnpreds],'Verbose',0, ...
    'IsObjectiveDeterministic',true, ...
    'AcquisitionFunctionName','expected-improvement-plus');
zbest = bestPoint(results);

%Train an optimized GAM using the zbest values.
Mdl_fancy = fitrgam(Krill_for_GAM,'TIGR',"PredictorNames",["chla","temperature"],...
    'CrossVal','on','verbose',1,...
```

```
    'MaxNumSplitsPerPredictor',zbest.maxNumSplits,
'NumTreesPerPredictor',zbest.numTrees,'InitialLearnRateForPredictors',zbest.learnpreds);
Mdl_F = fitrgam(Krill_for_GAM,'TIGR',"PredictorNames",["chla","temperature"],...
    'verbose',1,...
    'MaxNumSplitsPerPredictor',zbest.maxNumSplits,
'NumTreesPerPredictor',zbest.numTrees,'InitialLearnRateForPredictors',zbest.learnpreds);
yHat_fancy = kfoldPredict(Mdl_fancy);

%Now try the same thing but add apicomplexa moults perc as para4
minfunpara4 =
@(z)kfoldLoss(fitrgam(Krill_for_GAM,'TIGR',"PredictorNames",["chla","temperature","apicomplexa_
moults"],'CVPartition',c, ...
    'MaxNumSplitsPerPredictor',z.maxNumSplits, ...
    'NumTreesPerPredictor',z.numTrees, 'InitialLearnRateForPredictors',z.learnpreds));
rng('default')
resultspara1 = bayesopt(minfunpara4,[maxNumSplits,numTrees,learnpreds],'Verbose',0, ...
    'IsObjectiveDeterministic',true, ...
    'AcquisitionFunctionName','expected-improvement-plus');
zbestpara4 = bestPoint(resultspara1);
%Train an optimized GAM using the zbest values.
Mdl_fancy_para4 =
fitrgam(Krill_for_GAM,'TIGR',"PredictorNames",["chla","temperature","apicomplexa_moults"],...
    'CrossVal','on','verbose',1,...
    'MaxNumSplitsPerPredictor',zbestpara4.maxNumSplits,
'NumTreesPerPredictor',zbestpara4.numTrees,'InitialLearnRateForPredictors',zbestpara4.learnpreds);
Mdl_F_para4 =
fitrgam(Krill_for_GAM,'TIGR',"PredictorNames",["chla","temperature","apicomplexa_moults"],...
    'verbose',1,...
    'MaxNumSplitsPerPredictor',zbestpara4.maxNumSplits,
'NumTreesPerPredictor',zbestpara4.numTrees,'InitialLearnRateForPredictors',zbestpara4.learnpreds);
yHat_fancy_para4 = kfoldPredict(Mdl_fancy_para4);


%calculate the correlation cooefficients
GAM_corrcoef=zeros(2,5);
GAM_corrcoef(:,1)=[0, 4];
Corrcoef_matrix_0 = corrcoef(yHat_fancy,Krill_for_GAM.TIGR);
Corrcoef_matrix_4 = corrcoef(yHat_fancy_para4,Krill_for_GAM.TIGR);
GAM_corrcoef(1,2) = Corrcoef_matrix_0(1,2);
GAM_corrcoef(2,2) = Corrcoef_matrix_4(1,2);
GAM_corrcoef(:,3) = Corrcoef_matrix_0(1,2);
GAM_corrcoef(:,4) = GAM_corrcoef(:,2)-GAM_corrcoef(:,3);
%in the fifth column calculate Kfold loss
GAM_corrcoef(1,5) = kfoldLoss(Mdl_fancy);
GAM_corrcoef(2,5) = kfoldLoss(Mdl_fancy_para4);


%Make a figure
```

```
figure;
hold on
sgtitle('GAM with environment & Apicomplexa on moults')
subplot(2,4,1)
bars=bar(GAM_corrcoef(:,3:4),'stacked','FaceColor','flat');
bars(1).CData = Colour_env;
bars(2).CData = Colour_api_A;
title('Variation explained')
xticklabels({'Env only','Env + apicomplexa'})
subplot(2,4,2)
bars2=bar(GAM_corrcoef(:,5),'FaceColor','flat');
bars2.CData(1,:) = Colour_env;
bars2.CData(2,:) = Colour_api_A;
title('kfold loss (mean square error)')
ylim([36, 40])
xticklabels({'Env only','Env + apicomplexa'})
subplot(2,2,2)
plotPartialDependence(Mdl_F_para4,'chla')
subplot(2,2,3)
plotPartialDependence(Mdl_F_para4,'temperature')
subplot(2,2,4)
plotPartialDependence(Mdl_F_para4,'apicomplexa_moults')
hold off

%Note Partial dependence plot line colour adjusted in post-processing for
%simplicity.

%% %% Formally testing all possible apicomplexa metrics to confirm initial rough tests identified most meaningful predictor variable. There is probably a more efficient way to do this using loops, but this was faster to code

Krill_for_GAM=Krill_for_GAM_Oct13_2024(2:end,:);
c = cvpartition(length(Krill_for_GAM.TIGR),'KFold',50);
maxNumSplits = optimizableVariable('maxNumSplits',[1,20],'Type','integer');
numTrees = optimizableVariable('numTrees',[1,500],'Type','integer');
learnpreds = optimizableVariable('learnpreds',[0,0.25],'Type','real');

%Auto option optimizes these: InitialLearnRateForPredictors, NumTreesPerPredictor, Interactions, InitialLearnRateForInteractions, and NumTreesPerInteraction.

minfun =
@(z)kfoldLoss(fitrgam(Krill_for_GAM,'TIGR',"PredictorNames",["chla","temperature"],'CVPartition',c,
...
    'MaxNumSplitsPerPredictor',z.maxNumSplits, ...
    'NumTreesPerPredictor',z.numTrees, 'InitialLearnRateForPredictors',z.learnpreds));
rng('default')
results = bayesopt(minfun,[maxNumSplits,numTrees,learnpreds],'Verbose',0, ...
    'IsObjectiveDeterministic',true, ...
```

```
    'AcquisitionFunctionName','expected-improvement-plus');
zbest = bestPoint(results);
%Train an optimized GAM using the zbest values.
Mdl_fancy = fitrgam(Krill_for_GAM,'TIGR',"PredictorNames",["chla","temperature"],...
    'CrossVal','on','verbose',1,...
    'MaxNumSplitsPerPredictor',zbest.maxNumSplits,
'NumTreesPerPredictor',zbest.numTrees,'InitialLearnRateForPredictors',zbest.learnpreds);
Mdl_F = fitrgam(Krill_for_GAM,'TIGR',"PredictorNames",["chla","temperature"],...
    'verbose',1,...
    'MaxNumSplitsPerPredictor',zbest.maxNumSplits,
'NumTreesPerPredictor',zbest.numTrees,'InitialLearnRateForPredictors',zbest.learnpreds);
yHat_fancy = kfoldPredict(Mdl_fancy);

%Now try the same thing but add apicomplexa total bodies perc as para1
minfunpara1 =
@(z)kfoldLoss(fitrgam(Krill_for_GAM,'TIGR',"PredictorNames",["chla","temperature","apicomplexa_
bodies"],'CVPartition',c, ...
    'MaxNumSplitsPerPredictor',z.maxNumSplits, ...
    'NumTreesPerPredictor',z.numTrees, 'InitialLearnRateForPredictors',z.learnpreds));
rng('default')
resultspara1 = bayesopt(minfunpara1,[maxNumSplits,numTrees,learnpreds],'Verbose',0, ...
    'IsObjectiveDeterministic',true, ...
    'AcquisitionFunctionName','expected-improvement-plus');
zbestpara1 = bestPoint(resultspara1);
%Train an optimized GAM using the zbest values.
Mdl_fancy_para1 =
fitrgam(Krill_for_GAM,'TIGR',"PredictorNames",["chla","temperature","apicomplexa_bodies"],...
    'CrossVal','on','verbose',1,...
    'MaxNumSplitsPerPredictor',zbestpara1.maxNumSplits,
'NumTreesPerPredictor',zbestpara1.numTrees,'InitialLearnRateForPredictors',zbestpara1.learnpreds);
Mdl_F_para1 =
fitrgam(Krill_for_GAM,'TIGR',"PredictorNames",["chla","temperature","apicomplexa_bodies"],...
    'verbose',1,...
    'MaxNumSplitsPerPredictor',zbestpara1.maxNumSplits,
'NumTreesPerPredictor',zbestpara1.numTrees,'InitialLearnRateForPredictors',zbestpara1.learnpreds);
yHat_fancy_para1 = kfoldPredict(Mdl_fancy_para1);


%Now try the same thing but add apicomplexa total moults perc as para2
minfunpara2 =
@(z)kfoldLoss(fitrgam(Krill_for_GAM,'TIGR',"PredictorNames",["chla","temperature","apicomplexa_
moults"],'CVPartition',c, ...
    'MaxNumSplitsPerPredictor',z.maxNumSplits, ...
    'NumTreesPerPredictor',z.numTrees, 'InitialLearnRateForPredictors',z.learnpreds));
rng('default')
resultspara2 = bayesopt(minfunpara2,[maxNumSplits,numTrees,learnpreds],'Verbose',0, ...
    'IsObjectiveDeterministic',true, ...
    'AcquisitionFunctionName','expected-improvement-plus');
```

```
zbestpara2 = bestPoint(resultspara2);
%Train an optimized GAM using the zbest values.
Mdl_fancy_para2 =
fitrgam(Krill_for_GAM,'TIGR',"PredictorNames",["chla","temperature","apicomplexa_moults"],...
    'CrossVal','on','verbose',1,...
    'MaxNumSplitsPerPredictor',zbestpara2.maxNumSplits,
'NumTreesPerPredictor',zbestpara2.numTrees,'InitialLearnRateForPredictors',zbestpara2.learnpreds);
Mdl_F_para2 =
fitrgam(Krill_for_GAM,'TIGR',"PredictorNames",["chla","temperature","apicomplexa_moults"],...
    'verbose',1,...
    'MaxNumSplitsPerPredictor',zbestpara2.maxNumSplits,
'NumTreesPerPredictor',zbestpara2.numTrees,'InitialLearnRateForPredictors',zbestpara2.learnpreds);
yHat_fancy_para2 = kfoldPredict(Mdl_fancy_para2);

%Now try the same thing but add apicomplexa A bodies perc as para3
minfunpara3 =
@(z)kfoldLoss(fitrgam(Krill_for_GAM,'TIGR',"PredictorNames",["chla","temperature","Apicomplexa_
A_bodies"],'CVPartition',c, ...
    'MaxNumSplitsPerPredictor',z.maxNumSplits, ...
    'NumTreesPerPredictor',z.numTrees, 'InitialLearnRateForPredictors',z.learnpreds));
rng('default')
resultspara3 = bayesopt(minfunpara3,[maxNumSplits,numTrees,learnpreds],'Verbose',0, ...
    'IsObjectiveDeterministic',true, ...
    'AcquisitionFunctionName','expected-improvement-plus');
zbestpara3 = bestPoint(resultspara3);
%Train an optimized GAM using the zbest values.
Mdl_fancy_para3 =
fitrgam(Krill_for_GAM,'TIGR',"PredictorNames",["chla","temperature","Apicomplexa_A_bodies"],...
    'CrossVal','on','verbose',1,...
    'MaxNumSplitsPerPredictor',zbestpara3.maxNumSplits,
'NumTreesPerPredictor',zbestpara3.numTrees,'InitialLearnRateForPredictors',zbestpara3.learnpreds);
Mdl_F_para3 =
fitrgam(Krill_for_GAM,'TIGR',"PredictorNames",["chla","temperature","Apicomplexa_A_bodies"],...
    'verbose',1,...
    'MaxNumSplitsPerPredictor',zbestpara3.maxNumSplits,
'NumTreesPerPredictor',zbestpara3.numTrees,'InitialLearnRateForPredictors',zbestpara3.learnpreds);
yHat_fancy_para3= kfoldPredict(Mdl_fancy_para3);

%Now try the same thing but add Apicomplexa A moults as para4
minfunpara4 =
@(z)kfoldLoss(fitrgam(Krill_for_GAM,'TIGR',"PredictorNames",["chla","temperature","Apicomplexa_
A_moults"],'CVPartition',c, ...
    'MaxNumSplitsPerPredictor',z.maxNumSplits, ...
    'NumTreesPerPredictor',z.numTrees, 'InitialLearnRateForPredictors',z.learnpreds));
rng('default')
resultspara4 = bayesopt(minfunpara4,[maxNumSplits,numTrees,learnpreds],'Verbose',0, ...
    'IsObjectiveDeterministic',true, ...
    'AcquisitionFunctionName','expected-improvement-plus');
```

```
zbestpara4 = bestPoint(resultspara4);
%Train an optimized GAM using the zbest values.
Mdl_fancy_para4 =
fitrgam(Krill_for_GAM,'TIGR',"PredictorNames",["chla","temperature","Apicomplexa_A_moults"],...
   'CrossVal','on','verbose',1,...
   'MaxNumSplitsPerPredictor',zbestpara4.maxNumSplits,
'NumTreesPerPredictor',zbestpara4.numTrees,'InitialLearnRateForPredictors',zbestpara4.learnpreds);
Mdl_F_para4 =
fitrgam(Krill_for_GAM,'TIGR',"PredictorNames",["chla","temperature","Apicomplexa_A_moults"],...
   'verbose',1,...
   'MaxNumSplitsPerPredictor',zbestpara4.maxNumSplits,
'NumTreesPerPredictor',zbestpara4.numTrees,'InitialLearnRateForPredictors',zbestpara4.learnpreds);
yHat_fancy_para4 = kfoldPredict(Mdl_fancy_para4);


%Now try the same thing but add apicomplexa other bodies perc as para5
minfunpara5 =
@(z)kfoldLoss(fitrgam(Krill_for_GAM,'TIGR',"PredictorNames",["chla","temperature","Apicomplexa_
other_bodies"],'CVPartition',c, ...
   'MaxNumSplitsPerPredictor',z.maxNumSplits, ...
   'NumTreesPerPredictor',z.numTrees, 'InitialLearnRateForPredictors',z.learnpreds));
rng('default')
resultspara5 = bayesopt(minfunpara5,[maxNumSplits,numTrees,learnpreds],'Verbose',0, ...
   'IsObjectiveDeterministic',true, ...
   'AcquisitionFunctionName','expected-improvement-plus');
zbestpara5 = bestPoint(resultspara5);
%Train an optimized GAM using the zbest values.
Mdl_fancy_para5 =
fitrgam(Krill_for_GAM,'TIGR',"PredictorNames",["chla","temperature","Apicomplexa_other_bodies"]
,...
   'CrossVal','on','verbose',1,...
   'MaxNumSplitsPerPredictor',zbestpara5.maxNumSplits,
'NumTreesPerPredictor',zbestpara5.numTrees,'InitialLearnRateForPredictors',zbestpara5.learnpreds);
Mdl_F_para5 =
fitrgam(Krill_for_GAM,'TIGR',"PredictorNames",["chla","temperature","Apicomplexa_other_bodies"]
,...
   'verbose',1,...
   'MaxNumSplitsPerPredictor',zbestpara5.maxNumSplits,
'NumTreesPerPredictor',zbestpara5.numTrees,'InitialLearnRateForPredictors',zbestpara5.learnpreds);
yHat_fancy_para5 = kfoldPredict(Mdl_fancy_para5);


%Now try the same thing but add apicomplexa other moults perc as para6
minfunpara6 =
@(z)kfoldLoss(fitrgam(Krill_for_GAM,'TIGR',"PredictorNames",["chla","temperature","Apicomplexa_
other_moults"],'CVPartition',c, ...
   'MaxNumSplitsPerPredictor',z.maxNumSplits, ...
   'NumTreesPerPredictor',z.numTrees, 'InitialLearnRateForPredictors',z.learnpreds));
```

```
rng('default')
resultspara6 = bayesopt(minfunpara6,[maxNumSplits,numTrees,learnpreds],'Verbose',0, ...
    'IsObjectiveDeterministic',true, ...
    'AcquisitionFunctionName','expected-improvement-plus');
zbestpara6 = bestPoint(resultspara6);
%Train an optimized GAM using the zbest values.
Mdl_fancy_para6 =
fitrgam(Krill_for_GAM,'TIGR',"PredictorNames",["chla","temperature","Apicomplexa_other_moults"]
,...
    'CrossVal','on','verbose',1,...
    'MaxNumSplitsPerPredictor',zbestpara6.maxNumSplits,
'NumTreesPerPredictor',zbestpara6.numTrees,'InitialLearnRateForPredictors',zbestpara6.learnpreds);
Mdl_F_para6 =
fitrgam(Krill_for_GAM,'TIGR',"PredictorNames",["chla","temperature","Apicomplexa_other_moults"]
,...
    'verbose',1,...
    'MaxNumSplitsPerPredictor',zbestpara6.maxNumSplits,
'NumTreesPerPredictor',zbestpara6.numTrees,'InitialLearnRateForPredictors',zbestpara6.learnpreds);
yHat_fancy_para6 = kfoldPredict(Mdl_fancy_para6);


%calculate the correlation cooefficients
GAM_corrcoef=zeros(7,5);
GAM_corrcoef(:,1)=[0, 1, 2, 2, 4, 5, 6];
Corrcoef_matrix_0 = corrcoef(yHat_fancy,Krill_for_GAM.TIGR);
Corrcoef_matrix_1 = corrcoef(yHat_fancy_para1,Krill_for_GAM.TIGR);
Corrcoef_matrix_2 = corrcoef(yHat_fancy_para2,Krill_for_GAM.TIGR);
Corrcoef_matrix_3 = corrcoef(yHat_fancy_para3,Krill_for_GAM.TIGR);
Corrcoef_matrix_4 = corrcoef(yHat_fancy_para4,Krill_for_GAM.TIGR);
Corrcoef_matrix_5 = corrcoef(yHat_fancy_para5,Krill_for_GAM.TIGR);
Corrcoef_matrix_6 = corrcoef(yHat_fancy_para6,Krill_for_GAM.TIGR);
GAM_corrcoef(1,2) = Corrcoef_matrix_0(1,2);
GAM_corrcoef(2,2) = Corrcoef_matrix_1(1,2);
GAM_corrcoef(3,2) = Corrcoef_matrix_2(1,2);
GAM_corrcoef(4,2) = Corrcoef_matrix_3(1,2);
GAM_corrcoef(5,2) = Corrcoef_matrix_4(1,2);
GAM_corrcoef(6,2) = Corrcoef_matrix_5(1,2);
GAM_corrcoef(7,2) = Corrcoef_matrix_6(1,2);
GAM_corrcoef(:,3) = Corrcoef_matrix_0(1,2);
GAM_corrcoef(:,4) = GAM_corrcoef(:,2)-GAM_corrcoef(:,3);
%in the fifth column calculate Kfold loss
GAM_corrcoef(1,5) = kfoldLoss(Mdl_fancy);
GAM_corrcoef(2,5) = kfoldLoss(Mdl_fancy_para1);
GAM_corrcoef(3,5) = kfoldLoss(Mdl_fancy_para2);
GAM_corrcoef(4,5) = kfoldLoss(Mdl_fancy_para3);
GAM_corrcoef(5,5) = kfoldLoss(Mdl_fancy_para4);
GAM_corrcoef(6,5) = kfoldLoss(Mdl_fancy_para5);
GAM_corrcoef(7,5) = kfoldLoss(Mdl_fancy_para6);
```

```
%figure to compare the variation explained by different metrics of
%apicomplexa...
figure;
subplot(1,2,1)
bars=bar(GAM_corrcoef(:,3:4),'stacked','FaceColor','flat');
bars(1).CData = Colour_env;
bars(2).CData = Colour_api_A;
title('Variation explained')
ylim([-0.08, 0.28])
xticklabels({'Env only','Env + api tot bodies', 'Env + api tot moults', 'Env + api A bodies', 'Env + api A moults', 'Env + api other bodies', 'Env + api other moults'})
subplot(1,2,2)
bars2=bar(GAM_corrcoef(:,5),'FaceColor','flat');
bars2.CData(1,:) = Colour_env;
bars2.CData(2,:) = Colour_api_A;
bars2.CData(3,:) = Colour_api_A;
bars2.CData(4,:) = Colour_api_A;
bars2.CData(5,:) = Colour_api_A;
bars2.CData(6,:) = Colour_api_A;
bars2.CData(7,:) = Colour_api_A;
title('kfold loss (mean square error)')
ylim([36, 39])
xticklabels({'Env only','Env + api tot bodies', 'Env + api tot moults', 'Env + api A bodies', 'Env + api A moults', 'Env + api other bodies', 'Env + api other moults'})
%%
%Test out adding in interaction terms - one might expect parasites to have
%a greater impact under challenging conditions (e.g. food limitation or
%temperature extremes)

%Now try the same thing but add apicomplexa total bodies perc as para1
minfunpara1 =
@(z)kfoldLoss(fitrgam(Krill_for_GAM,'TIGR',"PredictorNames",["chla","temperature","apicomplexa_bodies"],'Interactions','all','MaxPValue',0.5,'CVPartition',c, ...
    'MaxNumSplitsPerPredictor',z.maxNumSplits, ...
    'NumTreesPerPredictor',z.numTrees, 'InitialLearnRateForPredictors',z.learnpreds));
rng('default')
resultspara1 = bayesopt(minfunpara1,[maxNumSplits,numTrees,learnpreds],'Verbose',0, ...
    'IsObjectiveDeterministic',true, ...
    'AcquisitionFunctionName','expected-improvement-plus');
zbestpara1 = bestPoint(resultspara1);
%Train an optimized GAM using the zbest values.
Mdl_fancy_para1 =
fitrgam(Krill_for_GAM,'TIGR',"PredictorNames",["chla","temperature","apicomplexa_bodies"],'Interactions','all','MaxPValue',0.5,...
    'CrossVal','on','verbose',1,...
    'MaxNumSplitsPerPredictor',zbestpara1.maxNumSplits,
'NumTreesPerPredictor',zbestpara1.numTrees,'InitialLearnRateForPredictors',zbestpara1.learnpreds);
```

```
Mdl_F_para1 =
fitrgam(Krill_for_GAM,'TIGR',"PredictorNames",["chla","temperature","apicomplexa_bodies"],'Intera
ctions','all','MaxPValue',0.5,...
   'verbose',1,...
   'MaxNumSplitsPerPredictor',zbestpara1.maxNumSplits,
'NumTreesPerPredictor',zbestpara1.numTrees,'InitialLearnRateForPredictors',zbestpara1.learnpreds);
yHat_fancy_para1 = kfoldPredict(Mdl_fancy_para1);


%Now try the same thing but add apicomplexa total moults perc as para2
minfunpara2 =
@(z)kfoldLoss(fitrgam(Krill_for_GAM,'TIGR',"PredictorNames",["chla","temperature","apicomplexa_
moults"],'Interactions','all','MaxPValue',0.5,'CVPartition',c, ...
   'MaxNumSplitsPerPredictor',z.maxNumSplits, ...
   'NumTreesPerPredictor',z.numTrees, 'InitialLearnRateForPredictors',z.learnpreds));
rng('default')
resultspara2 = bayesopt(minfunpara2,[maxNumSplits,numTrees,learnpreds],'Verbose',0, ...
   'IsObjectiveDeterministic',true, ...
   'AcquisitionFunctionName','expected-improvement-plus');
zbestpara2 = bestPoint(resultspara2);
%Train an optimized GAM using the zbest values.
Mdl_fancy_para2 =
fitrgam(Krill_for_GAM,'TIGR',"PredictorNames",["chla","temperature","apicomplexa_moults"],'Intera
ctions','all','MaxPValue',0.5,...
   'CrossVal','on','verbose',1,...
   'MaxNumSplitsPerPredictor',zbestpara2.maxNumSplits,
'NumTreesPerPredictor',zbestpara2.numTrees,'InitialLearnRateForPredictors',zbestpara2.learnpreds);
Mdl_F_para2 =
fitrgam(Krill_for_GAM,'TIGR',"PredictorNames",["chla","temperature","apicomplexa_moults"],'Intera
ctions','all','MaxPValue',0.5,...
   'verbose',1,...
   'MaxNumSplitsPerPredictor',zbestpara2.maxNumSplits,
'NumTreesPerPredictor',zbestpara2.numTrees,'InitialLearnRateForPredictors',zbestpara2.learnpreds);
yHat_fancy_para2 = kfoldPredict(Mdl_fancy_para2);


%Now try the same thing but add apicomplexa A bodies perc as para3
minfunpara3 =
@(z)kfoldLoss(fitrgam(Krill_for_GAM,'TIGR',"PredictorNames",["chla","temperature","Apicomplexa_
A_bodies"],'Interactions','all','MaxPValue',0.5,'CVPartition',c, ...
   'MaxNumSplitsPerPredictor',z.maxNumSplits, ...
   'NumTreesPerPredictor',z.numTrees, 'InitialLearnRateForPredictors',z.learnpreds));
rng('default')
resultspara3 = bayesopt(minfunpara3,[maxNumSplits,numTrees,learnpreds],'Verbose',0, ...
   'IsObjectiveDeterministic',true, ...
   'AcquisitionFunctionName','expected-improvement-plus');
zbestpara3 = bestPoint(resultspara3);
%Train an optimized GAM using the zbest values.
```

```
Mdl_fancy_para3 =
fitrgam(Krill_for_GAM,'TIGR',"PredictorNames",["chla","temperature","Apicomplexa_A_bodies"],'Int
eractions','all','MaxPValue',0.5,...
   'CrossVal','on','verbose',1,...
   'MaxNumSplitsPerPredictor',zbestpara3.maxNumSplits,
'NumTreesPerPredictor',zbestpara3.numTrees,'InitialLearnRateForPredictors',zbestpara3.learnpreds);
Mdl_F_para3 =
fitrgam(Krill_for_GAM,'TIGR',"PredictorNames",["chla","temperature","Apicomplexa_A_bodies"],'Int
eractions','all','MaxPValue',0.5,...
   'verbose',1,...
   'MaxNumSplitsPerPredictor',zbestpara3.maxNumSplits,
'NumTreesPerPredictor',zbestpara3.numTrees,'InitialLearnRateForPredictors',zbestpara3.learnpreds);
yHat_fancy_para3= kfoldPredict(Mdl_fancy_para3);


%Now try the same thing but add Apicomplexa A moults as para4
minfunpara4 =
@(z)kfoldLoss(fitrgam(Krill_for_GAM,'TIGR',"PredictorNames",["chla","temperature","Apicomplexa_
A_moults"],'Interactions','all','MaxPValue',0.5,'CVPartition',c, ...
   'MaxNumSplitsPerPredictor',z.maxNumSplits, ...
   'NumTreesPerPredictor',z.numTrees, 'InitialLearnRateForPredictors',z.learnpreds));
rng('default')
resultspara4 = bayesopt(minfunpara4,[maxNumSplits,numTrees,learnpreds],'Verbose',0, ...
   'IsObjectiveDeterministic',true, ...
   'AcquisitionFunctionName','expected-improvement-plus');
zbestpara4 = bestPoint(resultspara4);
%Train an optimized GAM using the zbest values.
Mdl_fancy_para4 =
fitrgam(Krill_for_GAM,'TIGR',"PredictorNames",["chla","temperature","Apicomplexa_A_moults"],'Int
eractions','all','MaxPValue',0.5,...
   'CrossVal','on','verbose',1,...
   'MaxNumSplitsPerPredictor',zbestpara4.maxNumSplits,
'NumTreesPerPredictor',zbestpara4.numTrees,'InitialLearnRateForPredictors',zbestpara4.learnpreds);
Mdl_F_para4 =
fitrgam(Krill_for_GAM,'TIGR',"PredictorNames",["chla","temperature","Apicomplexa_A_moults"],'Int
eractions','all','MaxPValue',0.5,...
   'verbose',1,...
   'MaxNumSplitsPerPredictor',zbestpara4.maxNumSplits,
'NumTreesPerPredictor',zbestpara4.numTrees,'InitialLearnRateForPredictors',zbestpara4.learnpreds);
yHat_fancy_para4 = kfoldPredict(Mdl_fancy_para4);



%Now try the same thing but add apicomplexa other bodies perc as para5
minfunpara5 =
@(z)kfoldLoss(fitrgam(Krill_for_GAM,'TIGR',"PredictorNames",["chla","temperature","Apicomplexa_
other_bodies"],'Interactions','all','MaxPValue',0.5,'CVPartition',c, ...
   'MaxNumSplitsPerPredictor',z.maxNumSplits, ...
```

```
    'NumTreesPerPredictor',z.numTrees, 'InitialLearnRateForPredictors',z.learnpreds));
rng('default')
resultspara5 = bayesopt(minfunpara5,[maxNumSplits,numTrees,learnpreds],'Verbose',0, ...
    'IsObjectiveDeterministic',true, ...
    'AcquisitionFunctionName','expected-improvement-plus');
zbestpara5 = bestPoint(resultspara5);
%Train an optimized GAM using the zbest values.
Mdl_fancy_para5 =
fitrgam(Krill_for_GAM,'TIGR',"PredictorNames",["chla","temperature","Apicomplexa_other_bodies"]
,'Interactions','all','MaxPValue',0.5,...
    'CrossVal','on','verbose',1,...
    'MaxNumSplitsPerPredictor',zbestpara5.maxNumSplits,
'NumTreesPerPredictor',zbestpara5.numTrees,'InitialLearnRateForPredictors',zbestpara5.learnpreds);
Mdl_F_para5 =
fitrgam(Krill_for_GAM,'TIGR',"PredictorNames",["chla","temperature","Apicomplexa_other_bodies"]
,'Interactions','all','MaxPValue',0.5,...
    'verbose',1,...
    'MaxNumSplitsPerPredictor',zbestpara5.maxNumSplits,
'NumTreesPerPredictor',zbestpara5.numTrees,'InitialLearnRateForPredictors',zbestpara5.learnpreds);
yHat_fancy_para5 = kfoldPredict(Mdl_fancy_para5);


%Now try the same thing but add apicomplexa other moults perc as para6
minfunpara6 =
@(z)kfoldLoss(fitrgam(Krill_for_GAM,'TIGR',"PredictorNames",["chla","temperature","Apicomplexa_
other_moults"],'Interactions','all','MaxPValue',0.5,'CVPartition',c, ...
    'MaxNumSplitsPerPredictor',z.maxNumSplits, ...
    'NumTreesPerPredictor',z.numTrees, 'InitialLearnRateForPredictors',z.learnpreds));
rng('default')
resultspara6 = bayesopt(minfunpara6,[maxNumSplits,numTrees,learnpreds],'Verbose',0, ...
    'IsObjectiveDeterministic',true, ...
    'AcquisitionFunctionName','expected-improvement-plus');
zbestpara6 = bestPoint(resultspara6);
%Train an optimized GAM using the zbest values.
Mdl_fancy_para6 =
fitrgam(Krill_for_GAM,'TIGR',"PredictorNames",["chla","temperature","Apicomplexa_other_moults"]
,'Interactions','all','MaxPValue',0.5,...
    'CrossVal','on','verbose',1,...
    'MaxNumSplitsPerPredictor',zbestpara6.maxNumSplits,
'NumTreesPerPredictor',zbestpara6.numTrees,'InitialLearnRateForPredictors',zbestpara6.learnpreds);
Mdl_F_para6 =
fitrgam(Krill_for_GAM,'TIGR',"PredictorNames",["chla","temperature","Apicomplexa_other_moults"]
,'Interactions','all','MaxPValue',0.5,...
    'verbose',1,...
    'MaxNumSplitsPerPredictor',zbestpara6.maxNumSplits,
'NumTreesPerPredictor',zbestpara6.numTrees,'InitialLearnRateForPredictors',zbestpara6.learnpreds);
yHat_fancy_para6 = kfoldPredict(Mdl_fancy_para6);
```

```
%Read out the interaction terms which are significant:
Mdl_F_para1.Interactions
Mdl_F_para2.Interactions
Mdl_F_para3.Interactions
Mdl_F_para4.Interactions
Mdl_F_para5.Interactions
Mdl_F_para6.Interactions


%% Apply some simplifying assumptions to calculate an estimate of how much biomass is lost to parasites through reduced growth at a circumpolar level

%Global biomass of E. superba is difficult to estimate - here using Angus's
%2009 estimate from krill base of 379 million tons (379*10^12 g), this is
%also reasonable in the context of other available estimates (based on
%Siegel & Watkins chapter in Biology & Ecology of Antarctic krill)

%The average krill is 41 mm long, based on table 1 in Tarling et al. 2016
%(MEPS)

%Morris et al. 1998 calculates wet weight of krill (grams) from length (mm)
%as weight=0.00000339*L^3.23

%Use the median intermoult period from Tarling et al. 2006 of 18 days

IMP_in_years=18/365;

%Calculate the number of krill in the ocean

Global_krill_length=41;
Global_krill_weight_per_indiv=0.00000339*Global_krill_length^3.23;
Total_number_krill=(379*10^12)/Global_krill_weight_per_indiv;

%For apicomplexa - the impact is observed as a step change, with TIGR (%
%change in length) dropping from 6.07 to 4.45 between Apicomplexa loads of 37 & 38% total
%sequence reads

Global_krill_weight_pg_no_api=0.00000339*(Global_krill_length*1.0607)^3.23;
Global_krill_weight_pg_api=0.00000339*(Global_krill_length*1.0445)^3.23;

%What perc of krill are above the api threshold?

Krill_with_api_perc=(sum(Krill_for_GAM.apicomplexa_moults>37))/(sum(isfinite(Krill_for_GAM.apicomplexa_moults)));

Global_new_biomass_no_api=(Global_krill_weight_pg_no_api - Global_krill_weight_per_indiv)*Total_number_krill;
```

Global_new_biomass_prop_api=(((Global_krill_weight_pg_no_api - Global_krill_weight_per_indiv)*Total_number_krill)*(1-Krill_with_api_perc))+(((Global_krill_weight_pg_api - Global_krill_weight_per_indiv)*Total_number_krill)*Krill_with_api_perc);

Biomass_diff_per_year_Mt_api=((Global_new_biomass_no_api-Global_new_biomass_prop_api)/IMP_in_years)*(1/10^12)

New_biomass_per_year_no_api=(Global_new_biomass_no_api/IMP_in_years)*(1/10^12);

Proportion_lost_to_api=Biomass_diff_per_year_Mt_api/New_biomass_per_year_no_api

%Now for ciliates. Line fit is: TIGR=-0.36(% Ciliates) + 5.92 (calculated using the fit toolbox pop up)

%average % ciliates

Ciliate_average=(sum(Krill_for_GAM.ciliates_cde_moults(isfinite(Krill_for_GAM.ciliates_cde_moults))))/(sum(isfinite(Krill_for_GAM.ciliates_cde_moults)));

Global_krill_weight_pg_no_cili=0.00000339*(Global_krill_length*(1+(5.92*(1/100))))^3.23;
Global_krill_weight_pg_cili=0.00000339*(Global_krill_length*(1+(5.92-0.36*Ciliate_average)*(1/100)))^3.23;

Global_new_biomass_no_cili=(Global_krill_weight_pg_no_cili - Global_krill_weight_per_indiv)*Total_number_krill;
Global_new_biomass_cili=(Global_krill_weight_pg_cili - Global_krill_weight_per_indiv)*Total_number_krill;

Biomass_diff_per_year_Mt_cili=((Global_new_biomass_no_cili-Global_new_biomass_cili)/IMP_in_years)*(1/10^12)

New_biomass_per_year_no_cili=(Global_new_biomass_no_cili/IMP_in_years)*(1/10^12);

Proportion_lost_to_cili=Biomass_diff_per_year_Mt_cili/New_biomass_per_year_no_cili

%If we take the conservative assumption that krill only grow November -
%February, that would lead to an overall impact of 1/3

Conservative_total_biomass_lost_to_parasites=(Biomass_diff_per_year_Mt_api+Biomass_diff_per_year_Mt_cili)/3
